# Identification and characteristics of microRNAs from *Bombyx mori*

**DOI:** 10.1186/1471-2164-9-248

**Published:** 2008-05-28

**Authors:** Ping-an He, Zuoming Nie, Jianqing Chen, Jian Chen, Zhengbing Lv, Qing Sheng, Songping Zhou, Xiaolian Gao, Lingyin Kong, Xiangfu Wu, Yongfeng Jin, Yaozhou Zhang

**Affiliations:** 1 Institute of Biochemistry, Zhejiang Sci-Tech University, Hangzhou 310018, PR China; 2 College of Science, Zhejiang Sci-Tech University, Hangzhou 310018, PR China; 3 Department of Biology and Biochemistry, University of Houston, Houston, Texas 77204-5001, USA; 4 Institute of Biochemistry, Zhejiang University, Hangzhou 310029, PR China

## Abstract

**Background:**

MicroRNAs (miRNAs) are small RNA molecules that regulate gene expression by targeting messenger RNAs (mRNAs) and causing mRNA cleavage or translation blockage. Of the 355 *Arthropod *miRNAs that have been identified, only 21 are *B. mori *miRNAs that were predicted computationally; of these, only *let-7 *has been confirmed by Northern blotting.

**Results:**

Combining a computational method based on sequence homology searches with experimental identification based on microarray assays and Northern blotting, we identified 46 miRNAs, an additional 21 plausible miRNAs, and a novel small RNA in *B. mori*. The latter, *bmo-miR-100-like*, was identified using the known miRNA *aga-miR-100 *as a probe; *bmo-miR-100-like *was detected by microarray assay and Northern blotting, but its precursor sequences did not fold into a hairpin structure. Among these identified miRNAs, we found 12 pairs of miRNAs and miRNA*s. Northern blotting revealed that some *B. mori *miRNA genes were expressed only during specific stages, indicating that *B. mori *miRNA genes (e.g., *bmo-miR-277*) have developmentally regulated patterns of expression. We identified two miRNA gene clusters in the *B. mori *genome. *bmo-miR-2b*, which is found in the gene cluster bmo-miR-2a-1/bmo-miR-2a-1*/bmo-miR-2a-2/bmo-miR-2b/bmo-miR-13a*/bmo-miR-13b, encodes a newly identified member of the *mir-2 *family. Moreover, we found that methylation can increase the sensitivity of a DNA probe used to detect a miRNA by Northern blotting. Functional analysis revealed that 11 miRNAs may regulate 13 *B. mori *orthologs of the 25 known *Drosophila *miRNA-targeted genes according to the functional conservation. We predicted the binding sites on the 1671 3'UTR of *B. mori *genes; 547 targeted genes, including 986 target sites, were predicted. Of these target sites, 338 had perfect base pairing to the seed region of 43 miRNAs. From the predicted genes, 61 genes, each of them with multiple predicted target sites, should be considered excellent candidates for future functional studies. Biological classification of predicted miRNA targets showed that "binding", "catalytic activity" and "physiological process" were over-represented for the predicted genes.

**Conclusion:**

Combining computational predictions with microarray assays, we identified 46 *B. mori *miRNAs, 13 of which were miRNA*s. We identified a novel small RNA and 21 plausible *B. mori *miRNAs that could not be located in the available *B. mori *genome, but which could be detected by microarray. Thirteen and 547 target genes were predicted according to the functional conservation and binding sites, respectively. Identification of miRNAs in *B. mori*, particularly those that are developmentally regulated, provides a foundation for subsequent functional studies.

## Background

MicroRNAs (miRNAs) are a class of endogenous, small, non-coding, single-stranded RNA. They comprise approximately 19–25 nucleotides that are embedded within the stem regions of hairpin transcripts (called "pre-miRNAs") [1-9]. The first miRNA genes, *lin-4 *[5] and *let-7 *[6], were identified in *Caenorhabditis elegans *about a decade ago. Many endogenously encoded miRNAs have been detected in mammals, plants, insects, worms, and viruses through cloning, Northern blotting, microarray assay, sequencing of short RNA molecules [1-9], and computation [10-20]. By December 2007, 5,395 hairpin sequence entries, containing 5,234 mature miRNAs, have been stored in miRBase [21-23]. Sequence analyses have shown that some mature miRNAs are phylogenetically conserved, particularly in the first 8 residues at the 5' end in species of the same kingdom (*let-7 *miRNAs are present in the human, mouse, rat, cow, dog, pig, chimpanzee, monkey, fish, and various insects, including *Bombyx mori *(*B. mori*)) [21]. In rare cases, mature miRNA sequences are conserved between animals and plants. For instance, *mir-854*, which is conserved in *C. elegans*, mouse, and human, has also been identified in plants [24]. However, hairpin sequences of precursor miRNAs are phylogenetically diverse. In addition to these characteristics, the genomic locations of miRNA precursor genes and the folding structures of miRNAs have been used to identify previously unknown miRNAs [10-20]. Lai *et al. *(2003) [11] stated that three characteristics allow miRNA genes to be identified using computational approaches: (i) miRNAs are generally derived from 70–100 nucleotide precursor transcripts having an extended stem-loop structure; (ii) miRNAs are usually conserved between genomes of related species; and (iii) miRNAs display a characteristic pattern of evolutionary divergence [10-20]. Additionally, genomic mapping of known miRNAs has enabled identification of orthologous miRNAs in other species (e.g., *B. mori*) where genomic annotations are lacking [25,26].

miRNAs are well-studied RNA interference (RNAi) molecules that silence gene expression of sequence-specific targeted messenger RNAs (mRNAs) in all species in which they have been found [27-29]. miRNAs silence gene expression by inducing miRNA-guided mRNA degradation, or by inhibiting translation by forming a miRNA-protein complex that binds to targeted mRNAs [1-4]. Some miRNAs target the non-coding region of a RNA gene transcript [29]. miRNAs are a class of small RNAs that have temporal and spatial specific expression patterns, and that have important roles in the development, proliferation, differentiation, transformation, and death of cells [30-33]. miRNAs affect cell fate during all stages of life, from renewable, pluripotent embryonic cells to fully differentiated cells; miRNAs can also affect aberrant cell growth in some tumors and carcinomas [32]. The ability of miRNAs to silence gene expression through a post-transcriptional mechanism has promoted their use as genetic tools in basic mechanistic studies; miRNAs are also being developed as biomarkers, therapeutic targets and therapeutic agents.

Based on computational cross-genome comparison predictions and experimental identifications, over 5,000 miRNAs have been identified in various organisms, including 152 miRNAs from *Drosophila melanogaster*, 73 from *Drosophila pseudoobscura*, 54 from *Apis mellifera*, 45 from *Anopheles gambiae*, 21 from *B. mori*, 137 from *C. elegans*, and 541 human miRNAs (miRBase release 10.1, December 2007). *B. mori *is a species of *Arthropod*, closely related to *Nematodes *and *Platyhelminthes*. MiRBase contains 355 miRNA genes in *Arthropods *(mosquito, honey bee and fruit flies), 232 in *Nematodes *(*C. elegans *and *C. briggsae*) and 63 in *Platyhelminthes *(*Schmidtea mediterranea*) [34]. Of the 355 *Arthropod *miRNAs identified, 21 are *B. mori *miRNAs that were predicted computationally by our research group [19]; of these, only *let-7 *has been confirmed by Northern blotting [35,36].

In this study, we report the first set of *B. mori *miRNA genes from larva, moth and pupa. We established our initial computational method for predicting miRNAs by genome-wide miRNA mapping using the available *B. mori *genomic sequences and *Arthropod *miRNAs. We further confirmed their identities using comprehensive microarray profiling and Northern blotting. miRNA targeted genes were predicted according to functional conservation and binding sites. Biological classification of predicted targets was determined by GO analysis.

## Results

### Prediction of *B. mori *miRNAs

Based on the sequences of all miRNAs previously identified in *Arthropoda*, we searched the *B. mori *genome by BLAST. According to the four criteria described in Materials and Methods, we identified 31 potential *B. mori *miRNAs and their precursor sequences. Information on predicted *B. mori *miRNAs, including names, lengths, source, and genomic position, are listed in Table 1. The length of the 31 predicted *B. mori *miRNAs ranged from 19 nt to 26 nt. We used the *mfold *program to fold each of the 31 predicted *B. mori *pre-miRNA sequences, ranging in length from 70 nt to 102 nt, and allowing G-U pairing, into hairpin structures [37]. The free energy of folding for these hairpin structures ranged from -24.3 kcal/mol to -48.4 kcal/mol.

**Table 1:** Information for all 46 predicted *B. mori *miRNA sequences

**miRNAs**	**Sequence**	**Size (nts)**	**miRBase/first prediction**	**Predicted in this study**	**Northern blot identification**	**Source**	**Position**	**Number of mismatched bp with *Drosophila *orthologs**	**Number of potential targets**
*bmo-let-7a*	GAGGUAGUAGGUUGUAUAGU	20	yes	yes	yes	BAAB01030021	359–379	0	10
bmo-let-7a*	ACUGUAUAGCCUGCUAACUUU	21	no	no	no	BAAB01030021	404–424		9
*bmo-miR-1*	UGGAAUGUAAAGAAGUAUGGAG	22	yes	yes	yes	BAAB01105211	2140–2161	0	1
bmo-miR-1*	UUCCGUGCUUCCUUACUUCCCA	22	no	no	no	BAAB01105211	2098–2124		12
*bmo-miR-2a-1*	UAUCACAGCCAGCUUUGAUGAGC	23	no	yes	yes	BAAB01090954	2892–2870	0	22
bmo-miR-2a-1*	GGCAUCAAAGUCGGUUUGUCAUA	23	no	no	no	BAAB01090954	2929–2907		15
*bmo-miR-2a-2*	UAUCACAGCCAGCUUUGAUGAGC	23	no	no	yes	BAAB01090954	2476–2454	0	22
*bmo-miR-2b*	UAUCACAGCCAGCUUUGUUGAGU	22	no	yes	yes	BAAB01090954	2355–2334	2	38
*bmo-miR-7*	GUAUGGAAGACUAGUGAUUUUGUUGU	26	yes	yes	no	BAAB01189120	2066–2091	0	6
*bmo-miR-8*	UAAUACUGUCAGGUAAAGAUGUC	23	yes	yes	yes	BAAB01175925	75–53	0	5
bmo-miR-8*	GCAUCUUACCGGGCAGCAUUA	21	no	no	yes	BAAB01175925	115–95		16
*bmo-miR-9*	UCUUUGGUUAUCUAGCUGUAUGA	22	yes	yes	yes	BAAB01140220	35–56	0	10
bmo-miR-9*	UCAUAAAGCUAGGUUACCGGAG	22	no	no	yes	BAAB01140220	69–90		6
*bmo-miR-10*	ACCCUGUAGAUCCGAAUUUGU	21	yes	yes	no	BAAB01187436	558–538	1	3
bmo-miR-10*	ACAAAUUCGGUUCUAGAGAGGU	22	no	no	no	BAAB01187436	524–503		6
bmo-miR-13a*	CCUGUCAAAGCGGCGGUGAAA	21	no	no	no	BAAB01090954	2777–2757		35
*bmo-miR-13b*	UAUCACAGCCAUUUUUGACGAGU	23	no	yes	yes	BAAB01090954	2615–2593	1	6
*bmo-miR-14*	UCAGUCUUUUUCUCUCUCCUA	21	yes	no	yes	BAAB01082886	457–437	0	8
*bmo-miR-31*	GGCAAGAAGUCGGCAUAGCUG	21	yes	yes	yes	BAAB01205265	3219–3239	3	14
*bmo-miR-34*	GGCAGUGUGGUUAGCUGGUUGUGUA	25	yes	yes	no	BAAB01147222	1437–1471	1	197
*bmo-miR-46*	UGUCAUGGAGUUGCUCUCUUUA	22	no	no	no	BAAB01025185	703–722	2	7
bmo-miR-46*	UGAAGAGAGCUAUCCGUCGACA	22	no	no	no	BAAB01025185	664–685		10
*bmo-miR-71*	UGAAAGACAUGGGUAGUGA	19	yes	no	no	BAAB01131802	2236–2218	0	6
*bmo-miR-79*	UUCAUAAAGCUAGAUUACCAAAGCAU	26	no	yes	no	BAAB01113362	535–510	1	2
*bmo-miR-87*	UGAGCAAACUUUCAGGUGUGU	21	no	yes	no	BAAB01164262	758–778	2	4
*bmo-miR-133*	UUGGUCCCCUUCAACCAGCUGU	22	no	yes	no	BAAB01208622	2993–3014	0	11
*bmo-miR-184*	ACUGGACGGAGAACUGAUAAGGGC	24	no	yes	no	BAAB01083504	1684–1661	0	7
*bmo-miR-210*	UUGUGCGUGUGACAGCGGCU	20	no	yes	no	BAAB01031372	2309–2290	0	95
*bmo-miR-263a*	AAUGGCACUGGAAGAAUUCAC	21	yes	yes	yes	BAAB01106279	2833–2853	2	4
*bmo-miR-263b*	CUUGGCACUGGGAGAAUUCACAG	23	yes	yes	no	BAAB01015424	804–782	0	19
bmo-miR-263b*	CGUGAAUUUCCCGAUGCCUUA	21	no	no	no	BAAB01015424	764–744		8
*bmo-miR-275*	AGUCAGGUACCUGAAGUAGCGCGCG	25	yes	yes	no	BAAB01118959	480–456	0	41
*bmo-miR-276a*	UAGGAACUUCAUACCGUGCUCU	22	no	yes	yes	BAAB01199848	358–337	0	1
bmo-miR-276a*	(C)AGCGAGGUAUAGAGUUCCUA(CG)	22	yes	no	no	BAAB01199848	395–374		6
*bmo-miR-277*	UAAAUGCACUAUCUGGUACGACA	23	yes	no	yes	BAAB01014298	12099–12121	0	4
*bmo-miR-279*	UGACUAGAUCCACACUCAU	19	yes	yes	yes	BAAB01102170	714–732	3	0
bmo-miR-279*	AUGAGUGGAGGUUUAGUGCA	20	no	no	no	BAAB01102170	679–698		37
*bmo-miR-281a*	ACUGUCAUGGAGUUGCUCUCUU	22	no	yes	yes	BAAB01025185	701–722	3	18
bmo-miR-281a*	AAGAGAGCUAUCCGUCGACAGU	22	no	no	yes	BAAB01025185	666–687	0	10
*bmo-miR-282*	UAGCCUCUCCUUGGCUUUGUCUG	23	yes	yes	no	BAAB01005896	688–710	4	32
*bmo-miR-283*	UAAAUAUCAGCUGGUAAUUCUGGG	24	yes	yes	no	BAAB01105947	466–443	0	4
*bmo-miR-305*	UGUACUUCAUCAGGUGCUCUGG	22	Yes	yes	no	BAAB01118959	386–365	0	16
bmo-miR-305*	CAGGCGCUUGUUGGAGUACACU	22	no	no	no	BAAB01118959	352–331		66
*bmo-miR-307*	CAUCACAACCUCCUUGAGUGAGCGAU	26	yes	yes	no	BAAB01178566	1478–1503	0	19
*bmo-miR-317*	AGUGAACACAGCUGGUGGUAUC	22	no	yes	no	BAAB01140584	492–471	1	21
*bmo-bantam*	UGAGAUCAUUGUGAAAGCUAAUU	23	no	no	yes	BAAB01089624	1355–1373	1	1
*bmo-miR-100-like*	AACCCGUAGAUCCGAACUUGUG	22	no	no	yes	BAAB01129141	1755–1776	0	5

### miRNA microarray assay

Using the predicted *B. mori *miRNAs as guides, we designed probes to hybridize to targets in total RNA isolated from *B. mori *pupae and moths. Probes were classified into one of three groups, i.e., those designed to detect: 1) the 31 predicted miRNAs and their opposite-strand miRNAs; 2) the 704 known miRNAs in miRBase, which consist of miRNAs from several species, including six worm species, *D. melanogaster *(78 probes), *D. pseudoobscura *(4 probes), *A. gambiae *(14 probes), *A. mellifera *(7 probes), *C. elegans *(114 probes), *C. briggsae *(39 probes), and human (448 probes); and 3) control sequences, including the 5S rRNA of *B. mori *and *D. melanogaster*. The chip contained 954 probes in total. Each probe was repeated three times on the chip to ensure assay reproducibility.

**Figure 1:**
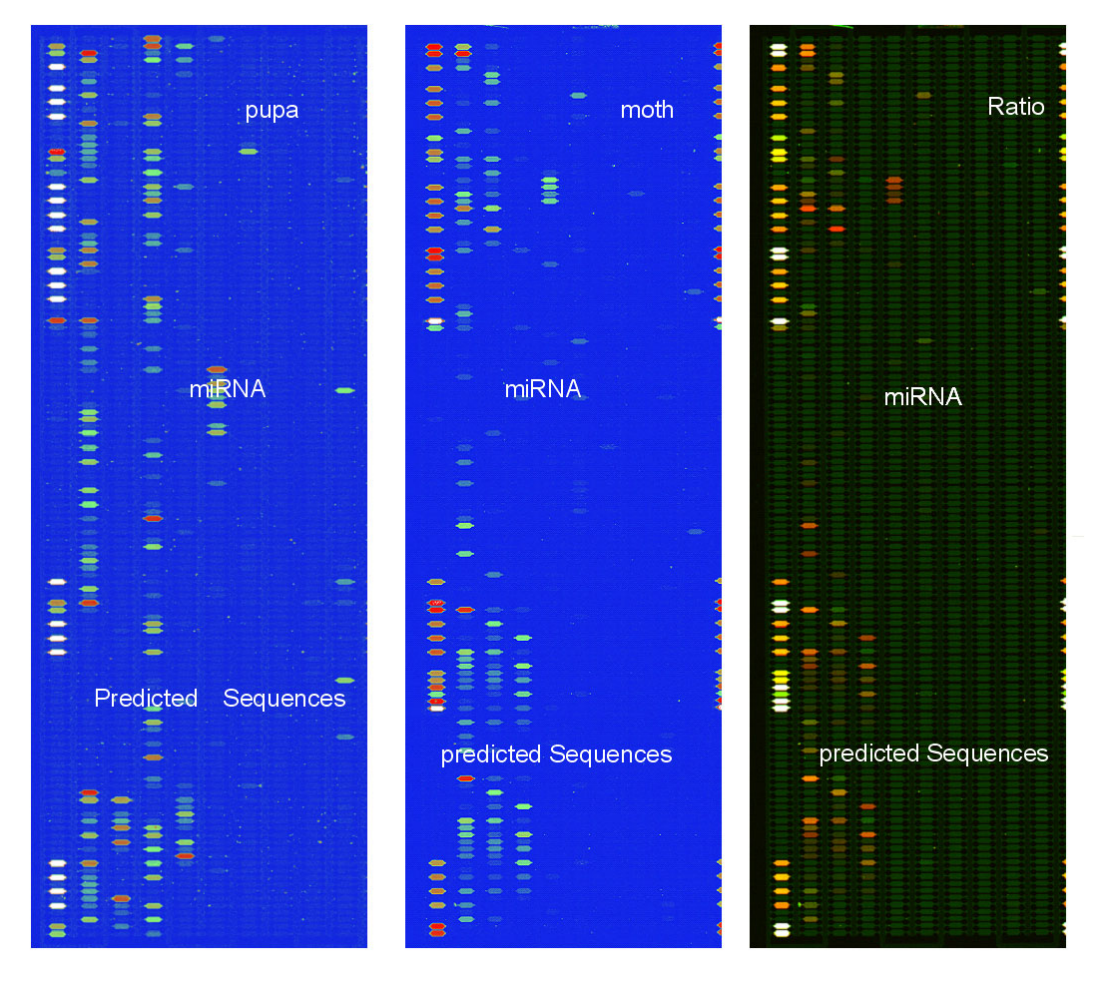
miRNA chip hybridization in *B. mori *pupa and moth.

**Figure 2:**
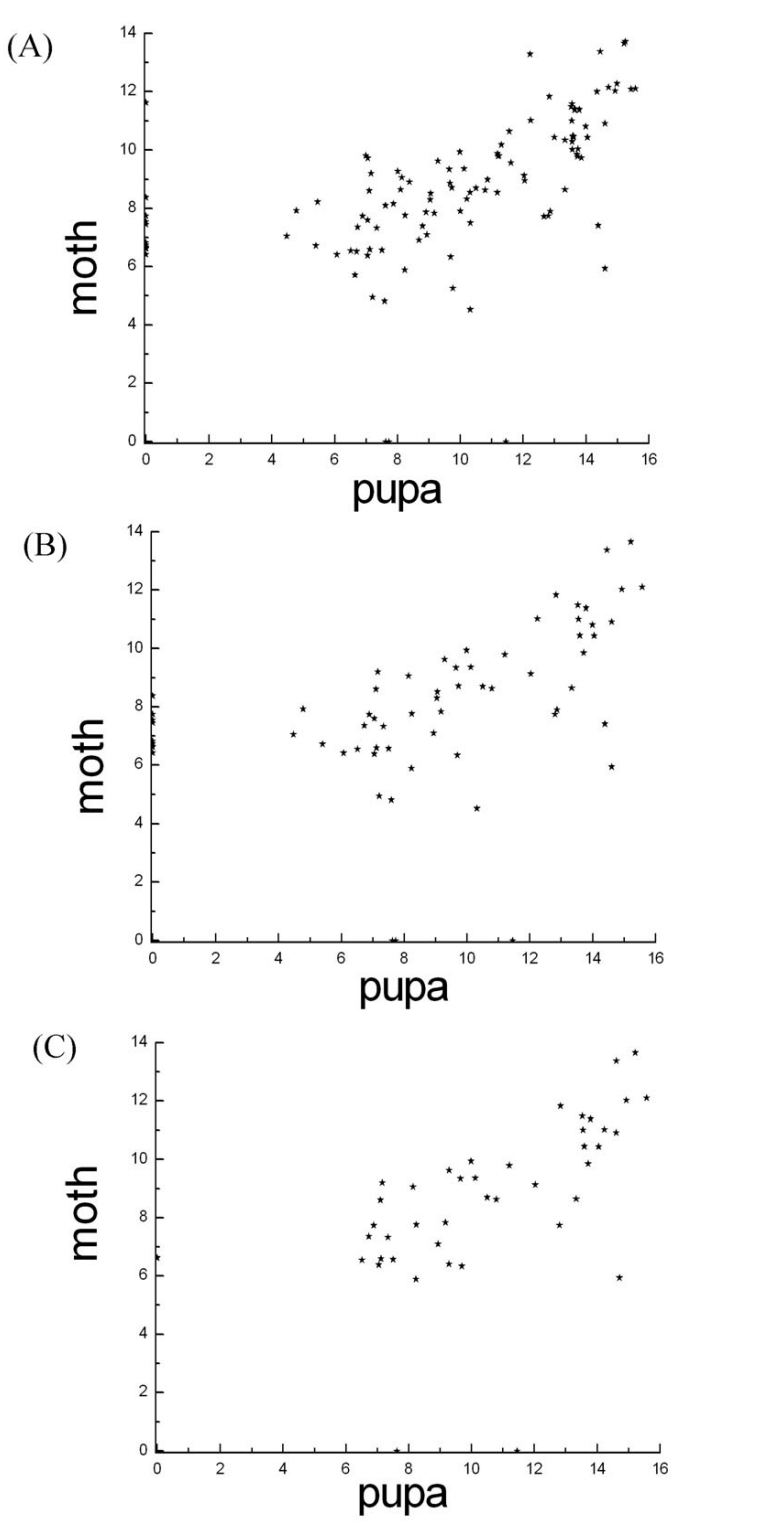
**Signal strength of detected miRNAs by microarray assay in *B. mori *pupa and moth.** a. all detected miRNAs; b. removed redundant miRNAs; c. *B. mori *miRNAs. N depicts the signal strength of detected miRNAs.

**Figure 3:**
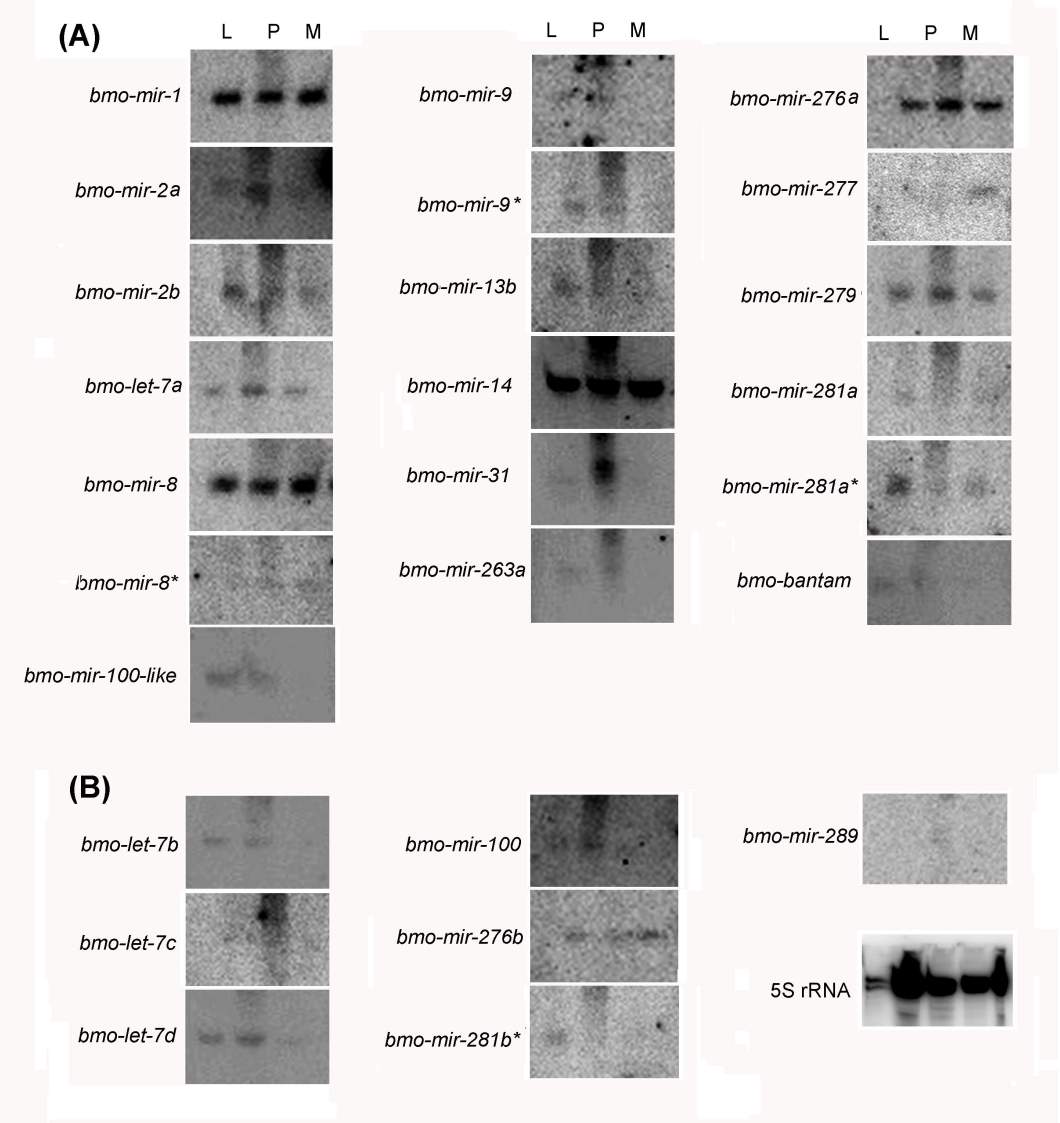
**Identification of miRNAs by Northern blotting using DNA probes complementary to the miRNA sequences.** (A). Northern blotting analysis of the miRNAs that belong to the group of 46 miRNAs identified by microarray assay, including the novel small RNA *bmo-miR-100-like*. (B). Northern blotting analysis of the miRNAs that belong to the group of 21 plausible *B. mori *miRNAs. The 5S rRNA band was the loading control. L, larva; P, pupa; M, moth.

Microarray results are shown in Figure 1. Comparison of the signal strengths for the miRNAs detected by microarray assays of RNA isolated from *B. mori *pupa and moth stages are shown in Figure 2. In most cases, results between spots for the same miRNA genes were very consistent in RNA isolated from *B. mori *pupa and moth (k = 0.908, b = 0.3104, R^2 ^= 0.8025). However, expression levels of some miRNAs exhibited obvious differences between the two developmental stages. Sequences exhibiting signal strengths of >80 were considered to be detected miRNA sequences. We detected 114 small RNAs from total RNA isolated from *B. mori *pupae and moths based on this cutoff. We applied a more stringent analysis by excluding miRNAs differing by only one single base in their sequences or by two bases near sequence ends, and identified 67 miRNAs and a novel small RNA. Of these 67 detected miRNAs, 27 miRNAs belonged to the 31 predicted miRNAs and 12 miRNAs were miRNA*s; another 28 miRNAs were detected only in the microarray assay using probes designed to detect known miRNA from other species. We used these 28 miRNA sequences as query sequences to perform BLAST searches against the *B. mori *genome. Seven of these sequences were identified in the *B. mori *genome and their precursor sequences could be folded into hairpin structures and, as such, these seven sequences were likely to be *B. mori *miRNAs. We designated these sequences bmo-miR-13a*, *bmo-miR-14*, *bmo-miR-46*, bmo-miR-46*, *bmo-miR-71*, *bmo-miR-277 *and *bmo-bantam*. Although *bmo-miR-13a *was one of the 31 miRNAs that were predicted, it was not detected by microarray assay, but its opposite-strand miRNA, named bmo-miR-13a*, was detected. The remaining 21 miRNAs were detected by microarray assay, but were not found in the available *B. mori *genome (possibly because sequencing of the *B. mori *genome is incomplete). We considered these 21 miRNAs to be plausible *B. mori *miRNAs. A novel small RNA was found using a probe designed to detect the known miRNA *aga-miR-100*. This small RNA was detected by microarray and Northern blotting (Figure 3A) and was identified in the *B. mori *genome, but its precursor sequence failed to fold into a hairpin structure. This novel small RNA was designated *bmo-miR-100-like*.

In conclusion, combining computational predictions with microarray assays, we identified 46 *B. mori *miRNAs, 13 of which were miRNA*s. We identified a novel small RNA and 21 plausible *B. mori *miRNAs that could not be found in the available *B. mori *genome, but which was detected by microarray. Detailed information for these 46 miRNAs and the novel small RNA are listed in Table 1. The 21 plausible *B. mori *miRNAs are listed in Table 1 of Additional file 1.

### Identification and determination of development-specific expression patterns of *B. mori *miRNAs by Northern blotting

Northern blotting was done based on microarray assay results to confirm the expression of *B. mori *miRNAs. Expression of the 46 identified miRNAs, the novel small RNA, and another 21 plausible miRNAs was investigated by Northern blotting of small-sized RNAs isolated from the larva, pupa, and moth stages of *B. mori*. Twenty-six miRNAs were stably expressed in *B. mori *in at least one developmental stage (Figure 3). Of these 26 miRNAs, 18 belong to the group of 46 miRNAs identified by microarray assay (Figure 3A) and 7 belong to the group of 21 plausible *B. mori *miRNAs (Figure 3B); the novel small RNA *bmo-miR-100-like *is shown in Figure 3A.

Development-specific expression patterns for some *B. mori *miRNAs were determined by Northern blotting. Some *B. mori *miRNA genes were expressed only in certain stages (Figure 3). *bmo-miR-1*, *bmo-let-7a*, *bmo-miR-8*, *bmo-miR-14*, *bmo-miR-276a*, *bmo-miR-279 *were strongly expressed in all developmental stages (larva, pupa and moth). They were uniformly expressed, suggesting that these miRNAs may play an important role in the regulation of some constitutive process *in B. mori*. Of these miRNAs, *bmo-miR-8 *has an opposite-strand miRNA, bmo-miR-8*, which was detected by Northern blotting (Figure 3A) at very low levels in larva, pupa and moth. *bmo-let-7b*, *bmo-let-7c*, bmo-miR-9, bmo-miR-9*, *bmo-miR-100-like*, *bmo-miR-263a*, *bmo-miR-31 *and *bmo-bantam *were expressed in larva and pupa, but were not detected in moth; of these miRNAs, *bmo-miR-9 *and bmo-miR-9* are also complementary miRNAs. *bmo-miR-281a*, bmo-miR-281a*, bmo-miR-281b*, *bmo-miR-13b *and *bmo-miR-2b *were expressed most strongly in larva, although they were also expressed in pupa and moth. *bmo-mri-2a*, *bmo-miR-100*, *bmo-miR-276b *and *bmo-let-7d *were also expressed in larva, pupa and moth; *bmo-mri-2a *and *bmo-miR-100 *were expressed most strongly in pupa; *bmo-miR-276b *was expressed most strongly in moth; *bmo-let-7d *was expressed most weakly in moth. *bmo-miR-277 *was expressed only in moth and not detected in larva and pupa; the precursor of *bmo-miR-277 *was also detected [see Figure S1 of Additional file 1]. Similarly, *bmo-miR-289 *was expressed weakly only in pupa and was not detected in larva and moth. Expression of a miRNA in a specific developmental stage may suggest a role for it in the developmental process.

### Computational prediction of *B. mori *miRNA targets

In the TarBase database, there are 23 miRNAs that regulate 34 targeted genes in *D. elanogaster*. miRNA functions could be evolutionally conserved between species such as *B. mori *and *D. melanogaster *[38]. To deduce the function of *B. mori *miRNAs, we searched for targeted genes of their orthologous miRNAs reported in *D. melanogaster *in the TarBase database [39]. In the 46 *B. mori *miRNAs identified, we found 11 miRNAs, belonging to 8 miRNA families; *Drosophila *orthologs of these 11 miRNAs have been reported in TarBase and are known to regulate the expression of at least 25 genes. These 11 miRNAs may regulate 13 *B. mori *orthologs of the 25 *Drosophila *miRNA-targeted genes according to binding [see Additional file 2]. *Bmo-miR-133 *may regulate two *B. mori *orthologs of *Mus musculus *miRNA targeted genes, SRF and Ptbp2 because of the perfect binding between miRNAs and the complementary sites. GO analysis showed that "nucleus" was over-represented for *Drosophila *orthologs of the 13 potential targeted genes.

We calculated the potential binding sites between miRNAs and the 3'UTR of mRNAs to determine the potential miRNA targeted genes more globally. With settings hybrid11 and hybridf22 described in Materials and Methods, we obtained 465 and 262 targeted genes, respectively. One hundred and eighty genes were involved in the two settings simultaneously. Removing redundancy, 547 targeted genes including 986 target sites were predicted. Of these binding sites, 338 had perfect base pairing to the seed region of 43 miRNAs. Of the 46 identified miRNAs, *bmo-miR-279 *was the only one for which we failed to find target sites. Certain miRNAs may have more than one target, and some predicted targets may be regulated by more than one miRNA [see Additional file 3 and Table 1]. Additionally, we found 61 3'UTRs that each contain multiple potential binding sites to a single miRNA [see Additional file 3]; these miRNA-mRNA duplexes showed higher specificity than others. Grun *et al*. (2005) predicted that at least15% of *D. melanogaste*r genes were regulated by at least one known miRNA [38]. Using settings hybrid11 (hybridf22), we found that 28% (16%) of annotated 1671 3'UTR sequences (corresponding to 1671 genes) had at least one binding site to miRNAs identified in this study. Compared with hybrid11, hybridf22 showed a significantly higher specificity. Biological classification of predicted miRNA targets was done by GO analysis to obtain insight into the function of *B. mori *miRNAs. GO analysis was completed by submitting gene sequences online (see Materials and Methods). In the predicted targets with settings hybrid11 (hybridf22), 360 (214) genes were assigned to the GO terms of "molecular function" ontology by BLAST, "binding" and "catalytic activity" of which over-represented for the 360 (214) genes; in the "biological process" ontology, "physiological process" over-represented among the 277(184) genes which were predicted with setting hybrid11 (hybridf22) and assigned to GO terms by BLAST [see Additional file 3].

## Discussion

We identified 46 miRNAs from *B. mori *using computational analyses and microarray assays. Of these 46 putative miRNAs, 18 were also confirmed by Northern blotting (Figure 3); although the remaining miRNAs were not detected by Northern blotting, they were considered to be miRNAs because they were detected by microarray and their precursors could adopt a hairpin structure. The reason why some miRNAs could not be detected by Northern blotting may be due to expression below the detection limit, or the very narrow temporal expression window for these miRNAs.

Methylated RNA probes have been employed by some research groups for RNA detection [40-42]. We found that methylation of the DNA probe used to detect miRNA by Northern blotting could increase sensitivity. Using methylated DNA probes, signal strength was significantly improved in Northern blots for *bmo-miR-14 *and bmo-miR-8*, which could not be detected using non-methylated DNA probes [see Figure S2 of Additional file 1]. Improvement may be due to the effect of methylation on base pairing of the probe with the target, or on the secondary structure of the probe.

### Analysis of *B. mori *miRNAs clusters

**Figure 4:**
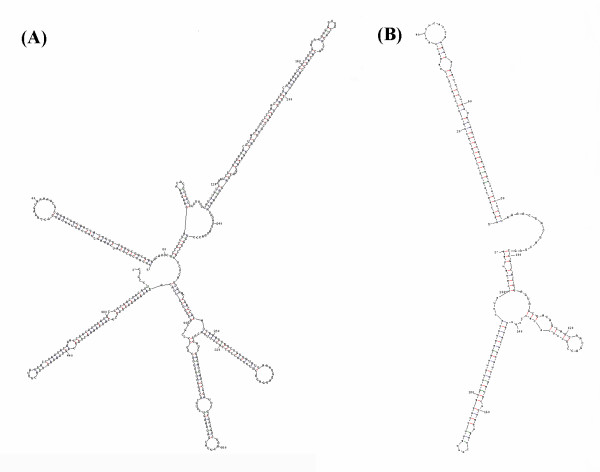
**Secondary structures of miRNA clusters.** a. bmo-miR-2a-1/bmo-miR-2a-1*/bmo-miR-2a-2/bmo-miR-2b/bmo-miR-13a*/bmo-miR-13b; b. bmo-miR-275/bmo-miR-305/bmo-miR-305*.

**Figure 5:**
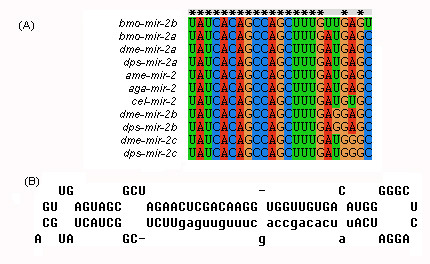
**Homologous analysis of *mir-2 *family.***bmo-miR-2b *is considered to be a new member of the *mir-2 *family. (A), Multi-alignment of *mir-2 *family miRNAs. (B), Hairpin structure of the precursor of *bmo-miR-2b*.

miRNA clusters have been reported in many species. A cluster usually contains two or three miRNA genes, although larger clusters have also been identified, such as the six-member *hsa-miR-17 *cluster [7,43,44] or the *D. melanogaster *cluster containing eight miRNA genes [45]. Clustered miRNA genes can share a high degree of similarity in nucleotide composition, but occasionally the miRNA sequences differ significantly [30,46]. Expression profiles for clustered genes are also very similar, raising the possibility that transcription of these miRNAs is controlled by a common regulatory element [47]. Examining the positions of the identified miRNAs in the *B. mori *genome, we identified two miRNA clusters (Figure 4): bmo-miR-2a-1/bmo-miR-2a-1*/bmo-miR-2a-2/bmo-miR-2b/bmo-miR-13a*/bmo-miR-13b; and bmo-miR-275/bmo-miR-305/bmo-miR-305*. The lengths of the two cluster sequences were 498 and 208 bp, respectively. Genes in the cluster containing bmo-miR-2a-1/bmo-miR-2a-1*/bmo-miR-2a-2/bmo-miR-2b/bmo-miR-13a*/bmo-miR-13b are members of the *mir-2 *miRNA family. This cluster contains *bmo-miR-2a*, which can be derived from two different precursor miRNAs transcribed from two paralogous miRNA genes, *bmo-miR-2a-1 *and *bmo-miR-2a-2*. The opposite strand to *bmo-miR-2a-1 *also encodes a miRNA. Searching miRBase, we found that the *bmo-miR-2a *sequence was identical to the *dme-miR-2a*, *dps-miR-2a*, *ame-miR-2*, and *aga-miR-2 *sequences, but we failed to identify any other miRNA that had the same sequence as *bmo-miR-2b *(Figure 5); therefore, *bmo-miR-2b *is a newly identified member of the *mir-2 *miRNA family. Searching the *D. melanogaster *and *A. gambiae *miRNAs, we found that corresponding *mir-2 *and *mir-13 *family members also assembled into clusters in these two species. *mir-2 *and *mir-13 *repress translation of the target genes, *grim, skl *and *rpr*, suggesting that they may be involved in regulating apoptosis [48]. The cluster bmo-miR-275/bmo-miR-305/bmo-miR-305* is also found in *D. melanogaster, D. pseudoobscura *and *A. gambiae*. *bmo-miR-275 *and *bmo-miR-305 *belong to different miRNA families, *mir-275 *and *mir-305*. The functions of these two miRNA families remain unknown.

### Analysis of *B. mori *miRNAs

The identified 46 *B. mori *miRNAs belonged to 29 miRNA families based on sequence similarity (Table 2). Among the 46 identified miRNAs, we found 12 pairs of miRNAs and miRNA*s. Scanning miRBase, we determined that seven of these 12 miRNA*s had not been reported. These newly identified miRNAs are bmo-miR-2a*, bmo-miR-8*, bmo-miR-13a*, bmo-miR-46*, bmo-miR-263*, bmo-miR-279*, and bmo-miR-305*.

**Table 2:** Comparisons of sequence similarity between *B. mori *miRNAs and corresponding miRNA family members

Index	Family	Mature		Pre-miRNA	
			
		Count	Similarity	Count	Similarity
1	*bantam*	5	>91% a	5	***
2	*let-7*	72	>80% a	101	*
3	*mir-1*	14	>86% a	17	**
4	*mir-2*	17	>92% a	16	**
5	*mir-7*	25	>85%	41	*
6	*mir-8*	5	100%	5	*
7	*mir-9*	29	>83% a	48	
8	*mir-10*	30	>86% a	34	**
9	*mir-14*	4	100%	9	*
10	*mir-31*	17	>81%	18	**
11	*mir-34*	31	>80% a	34	*
12	*mir-46*	3	>90%	3	*
13	*mir-71*	3	100%	3	***
14	*mir-79*	6	>90%	6	*
15	*mir-87*	5	>92%	5	**
16	*mir-133*	33	>88% a	37	*
17	*mir-184*	17	>90% a	18	**
18	*mir-210*	12	>90% a	12	**
19	*mir-263*	8	>80%	8	**
20	*mir-275*	4	100%	4	***
21	*mir-276*	6	>91% a	7	*
22	*mir-277*	5	100%	5	***
23	*mir-279*	4	100%	4	***
24	*mir-281*	5	>83%	7	*
25	*mir-282*	5	>85%	5	*
26	*mir-283*	4	100%	4	**
27	*mir-305*	5	100%	5	**
28	*mir-307*	4	100%	4	*
29	*mir-317*	5	>92% a	5	**

family: the name of miRNA family; count: the number of miRNAs in other species in the same miRNA family; similarity: the similarity between the sequences of *B. mori *miRNAs and the sequences of other members of the same family; the letter "a" denotes that this miRNA is identical to other mature miRNAs in the family; * denotes that this miRNA is only highly conserved in the miRNA-encoding region; ** denotes that this miRNA is highly conserved in the miRNA-encoding region and its opposite-strand region (miRNA*-encoding region); *** ** denotes that this miRNA is highly conserved in the precursor sequences.

Observing the orthologous miRNA genes for each family in miRBase, we found that 15 of the 29 families are exclusive to *Arthropoda*; the other 12 families are found in many species, including five species of *Arthropoda*. miRNAs having the most orthologs are *mir-133 *and *mir-9*, which are found in 25 and 23 animal species, including *D. melanogaster *and *C. elegans*.

We determined the number of orthologous and paralogous genes for each detected miRNA. The *let-7 *miRNA family contains the most members; 77 *let-7 *family members were identified in miRBase [see Figure S3 of Additional file 1].

### Analysis of *B. mori *precursor miRNA sequences

Variations in pre-miRNA sequences are related to their evolutionary history [22], so we compared the sequences of *B. mori *pre-miRNAs and members of the same family of pre-miRNAs in the miRBase database (Table 2). The number of the pre-miRNAs is greater than the number of mature miRNAs because mature miRNAs can be derived from two or more pre-miRNAs. This is consistent with the literature: mature miRNAs are conserved, but pre-miRNAs are diverse [23].

Members of the *bantam*, *mir-71, mir-275, mir-277, mir-279 *miRNA families have a high degree of similarity in their precursor sequences. *bmo-miR-279 *and other *mir-279 *precursor sequences have sequence similarities of >55%; *bmo-miR-277 *precursors share >56% sequence similarity; *bmo-miR-27 *precursors share >57% sequence similarity; *bmo-miR-71 *precursors share >59% sequence similarity; and *bmo-bantam *precursors share >60% sequence similarity.

**Figure 6:**
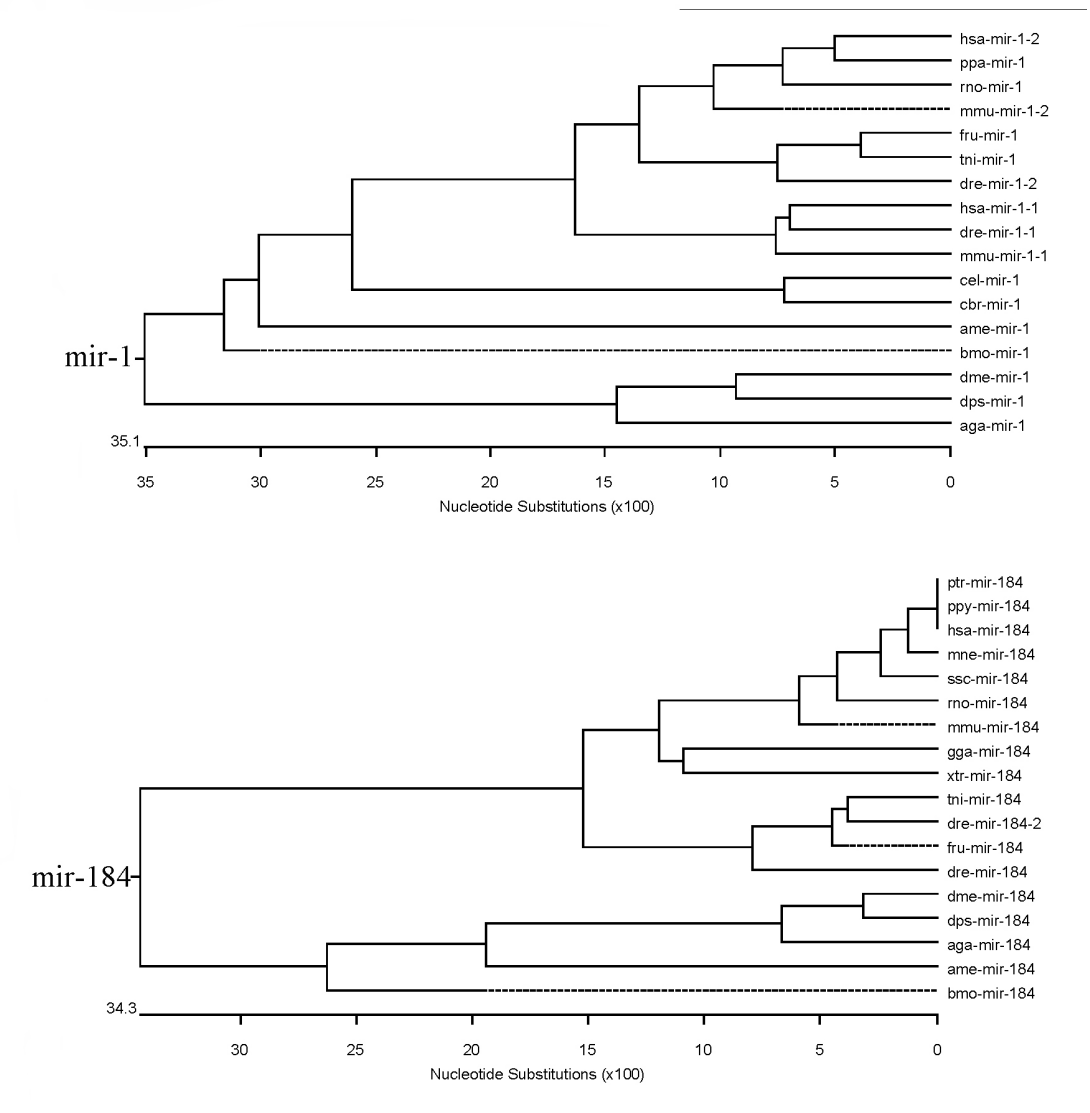
Phylogenetic tree of the *mir-1 *family and the *mir-184 *family.

Based on the results of our sequence comparisons, we determined the phylogenetic tree for each miRNA family using the DNAStar MegAlign program to analyze evolutionary relationships between miRNA members from each family. Many of the *B. mori *miRNA families were found only in *Arthropoda*; their mature and pre-miRNAs are highly conserved. For these miRNAs, we found that these *B. mori *miRNAs have close evolutionary relationships with *A. mellifera*, and *A. gambiae *miRNAs. We found that these *B. mori *miRNAs are located on the same branches of the phylogenetic tree as related miRNAs from *D. melanogaster*, *D. pseudoobscura*, *A. mellifera*, and *A. gambiae*. The phylogenetic tree for the *mir-1 *and *mir-184 *miRNA family is shown in Figure 6; other detailed results are listed in Additional file 4.

**Figure 7:**
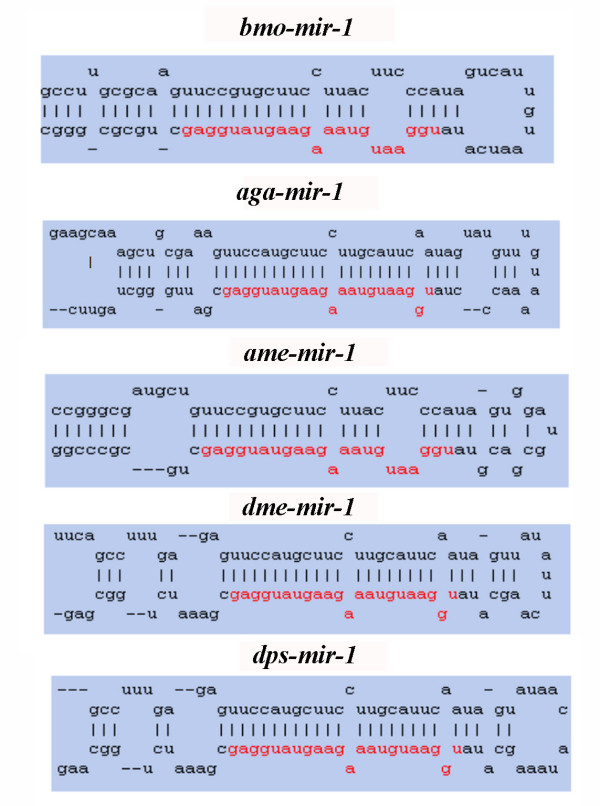
Comparison of the secondary structures of *bmo-miR-1*, *aga-miR-1*, *ame-miR-1*, *dme-miR-1 *and *dps-miR-1*.

We compared the hairpin structures of *B. mori *miRNA precursor sequences to those of related *D. melanogaster, D. pseudoobscura*, *A. mellifera*, and *A. gambiae *miRNA precursor sequences. Conserved regions of the precursors are found in two arms of the stem-loop structure. The results of comparisons between the hairpin structures of *bmo-miR-1, aga-miR-1, ame-miR-1, dme-miR-1 *and *dps-miR-1 *precursors are shown in Figure 7. Additional file 5 shows the secondary structures of *B. mori *miRNAs. These results strongly indicate that the identified 46 *B. mori *sequences are miRNAs.

### Computational prediction of *B. mori *miRNA targets

MiRNA can regulate the protein expression of genes based on the level of complementarity between miRNA seed sequences and binding sites on target mRNA [49-51]. For animals, the seed sequences of miRNA can bind to complementary sites on 3'UTR of its targeted gene. Complementary sites in targeted genes and seed sequences of miRNAs may be conserved in various species; this may result in the functional conservation of miRNAs, and the orthologous miRNAs may regulate orthologs of the targeted genes. The prediction for miRNA targeted genes according to functional conservation may offer additional insights into the function of miRNAs. Grun *et al. *(2005) computed the conserved regulatory microRNA-mRNA relationships [38]. They found that 50 unique gene pairs were predicted to be targeted by homologous microRNAs, including 60 microRNA-mRNA regulatory relationships [38]. Weaver *et al. *(2007) demonstrated that some microRNAs function in the same or similar way in *Drosophila *and bee (e.g., *mir-9a *may control sensory organ precursors (SOPs) between *Drosophila *and bee) [52]. Functional prediction based on cross-species conservation may provide a reference for studying on the functions of *B. mori *miRNAs. Based on this method, we identified 12 miRNAs that regulate 15 potential targeted genes [see Additional file 2]. Most *Drosophila *orthologs of these potential targeted genes were the vital genes for development, including transcriptional regulator, apoptosis-related genes and genes involved in signal transduction pathways. *mir-2 *controls HLHmdelta, and *mir-7 *controls HLHm3, HLHm5, HLHmgamma, M4 and TOM, in *D. melanogaster*; these six genes are involved in the Notch signaling pathway. *B. mori *homologs [GenBank: Bmo.1984 and Bmo.4220] of these six genes can bind perfectly to *mir-2 (bmo-miR-2a*, *bmo-miR-2b*, bmo-miR-13a* and *bmo-miR-13b*) and *bmo-miR-7*, respectively. *mir-2 *and *mir-7 *may be related to the Notch signaling pathway in *B. mori*; this hypothesis is consistent with previous predictions [38,53]. Cross-species comparisons allow for the identification of evolutionarily conserved and probable functional target sites. The 12 miRNAs could play parts in the development of *B. mori *by regulating their targeted genes, including the 15 potential targeted genes identified in this study.

We predicted many miRNA targeted genes by calculating target sites. Interestingly, we have studied *B. mori profilin *gene [GenBank: EF112402]; there was not a linear relationship between the transcription level of this gene and protein expression (data not shown). The long 3'UTR of this gene was found to have nine potential binding sites [see Additional file 3]. Profilin plays an important part in biologically active cellular compartments [54]. It regulates the polymerization, depolymerization, and dynamics of actin, which are important for determining cell shape and movement through the cytoplasm [54].

We can predict miRNA targets specifically according to the level of complementarity between the seed region of miRNA and 3'UTR of mRNA [49]. We set stringent settings for base pairing of binding sites (see Materials and Methods) based on this knowledge. Applying these settings, many targets predicted by functional conservation, such as *B. mori *homologs of HLHmdelta and M4, would not have been predicted because of stringent filtering of the settings for miRNA-mRNA duplexes. However, using the settings, the predicted binding sites of targets can bind perfectly to miRNA, and the target sites belong mainly to the three categories of known miRNA target sites classified by Sethupathy *et al. *(2006)[49] [see Additional file 3]. Nevertheless, we identified too many potential binding sites to test experimentally. According to a previous suggestion [49], genes with multiple predicted target sites (61 genes here accounting for 11% of all predicted targets) should be considered strong candidates for future functional studies.

## Conclusion

We identified 46 *B. mori *miRNAs, 13 of which were miRNA*s, by combining computational predictions with microarray assays. We identified a novel small RNA and 21 plausible *B. mori *miRNAs that could not be found in the available *B. mori *genome, but which were detected by microarray. The small novel RNA, *bmo-miR-100-like*, was identified using the known miRNA *aga-miR-100 *as a probe; it was detected by microarray assay and Northern blotting, but its precursor sequences failed to fold into a hairpin structure. Northern blotting revealed that some *B. mori *miRNA genes were expressed only during specific stages, indicating that *B. mori *miRNA genes have developmentally regulated expression patterns. For example, *bmo-miR-277 *was expressed only during the moth stage. We identified two miRNA gene clusters in the *B. mori *genome, and *bmo-miR-2b *was identified as a new member of the *mir-2 *family. We found that methylation of the DNA probe used to detect the miRNA by Northern blotting may increase its sensitivity. According to functional conservation in various species, we predicted that 11 miRNAs may regulate 13 *B. mori *orthologs of the 25 known *Drosophila *miRNA-targeted genes. A total of 547 targeted genes were predicted from 1671 *B. mori *genes according to the target sites predicted by settings hybrid11 and hybridf22. From the predicted genes, 61 genes each contained multiple predicted target sites and could be candidates for the functional study of miRNA. Three GO terms, "binding", "catalytic activity" and "physiological process", were over-represented for the predicted genes.

Identification of putative miRNAs in other organisms is a worthwhile approach for increasing the understanding of the roles of miRNA. Identification of miRNAs in *B. mori *may help to elucidate the mechanisms underlying simple and complex regulation of the 42-day lifecycle of *B. mori*, which consists of six periods of ecdysis and four periods of metamorphosis.

## Methods

### miRNA reference sets and the *B. mori *genome

**Figure 8:**
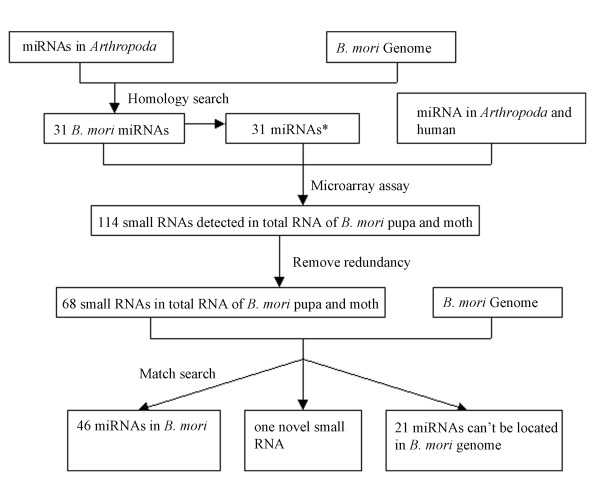
Predictions and microarray assays for miRNA in *B. mori*.

Prediction and identification of miRNAs in *B. mori *is shown in Figure 8. We downloaded 78 miRNAs from *D. melanogaster*, 73 from *D. pseudoobscura*, 25 from *A. mellifera*, 38 from *A. gambiae*, and their precursor sequences from MicroRNA Registry release 7.1 [21,22,55]. These sequences were used as a reference set to predict miRNA in *B. mori*. Genomic sequences for *B. mori *were downloaded from the Beijing Genomics Institute [25,26,56]. The total size of *B. mori *genome is 428.7 Mb, containing 23,156 scaffolds and 66,482 contigs.

### Prediction of *B. mori *miRNAs by homology search

We used the sequences of known miRNAs (*vide supra*) and pre-miRNAs in the reference set as query sequences for BLAST searches against the *B. mori *genome; we used a word size of seven and an E-value cutoff of ten.

Four criteria were used to predict *B. mori *miRNAs and pre-miRNAs according to BLAST search results: (1) there was >80% similarity between each potential *B. mori *miRNA and the corresponding miRNA in the reference set; (2) the difference in the lengths between each potential *B. mori *pre-miRNA and the corresponding pre-miRNA in reference set was <5-nt, and the corresponding positions of the mature miRNAs in their pre-miRNAs were nearly identical; (3) the lowest free energy for folding of the secondary structures for each potential *B. mori *pre-miRNA, as predicted by the *mfold *program [37], was less than -20 kcal/mol; and (4) the secondary structures of the predicted pre-miRNA satisfied the requirements set out in reference [23], i.e., a potential fold-back precursor structure must contain the ~22-nt miRNA sequence within one arm of the hairpin, and the hairpin must include at least 16 bp within the first 22 nt of the miRNA. The structure should not contain large internal loops or bulges, particularly not large asymmetric bulges (<5-nt).

### Paraflo™ miRNA microarray assay

Based on the predicted results and the sequences of known miRNAs in *Arthropoda*, *Nematoda*, and human, we designed probes to detect *B. mori *miRNAs in *B. mori *pupa and moth stages by microarray profiling.

Microarray assays were done using a service provider (LC Sciences, Houston, USA). The assay was done on 5-μg total RNA samples from *B. mori *pupa (labeled cy3) and moth (labeled cy5), which were size fractionated using a mirVana Isolation kit (Ambion, Austin, USA); the small RNAs (<200 nt) were 3'-extended with a poly(A) tail using poly(A) polymerase. An oligonucleotide tag was then ligated to the poly(A) tail for later staining with fluorescent dye; two different tags were used for the two RNA samples in dual-sample experiments. Hybridization was done overnight on a μParaFlo microfluidic chip using a micro-circulation pump (Atactic Technologies, Houston, USA) [57]. On the microfluidic chip, each detection probe consisted of a chemically modified nucleotide coding segment complementary to the target miRNA (miRBase, ) or other RNA (control or our defined sequences) and a spacer segment of polyethylene glycol to extend the coding segment away from the substrate. Detection probes were made by *in situ *synthesis using photogenerated reagent (PGR) chemistry. The hybridization melting temperatures were balanced by chemical modifications of the detection probes. Hybridizations used 100 L 6 × SSPE buffer (0.90 M NaCl, 60 mM Na_2_HPO_4_, 6 mM EDTA, pH 6.8) containing 25% formamide at 34°C. After hybridization, signals were detected using fluorescence labeling with tag-specfic Cy5 (for *B. mori *pupa and moth) dyes (Invitrogen, Carlsbad, USA). Hybridization images were collected using a laser scanner (GenePix 4000B, Molecular Device, Sunnyvale, USA) and quantified. Data were analyzed by first subtracting the background, and then normalized using a cyclic LOWESS filter (locally-weighted regression) [58]. For two color experiments, the ratio of the two sets of detected signals (log2 transformed, balanced) and p-values of the *t*-test were calculated; differentially detected signals were those with p-values of < 0.01. Data classification involved a hierarchical clustering method using average linkage and Euclidean distance metric, and was visualized with TIGR's MeV (Multiple Experimental Viewer; Institute for Genomic Research).

### Northern blotting

Total small RNA was isolated from the larva, pupa, and moth stages of the silkworm using mirVana™ miRNA Isolation Kit (Ambion) according to manufacturer instructions. Liquid nitrogen was used to freeze samples before RNA extraction. Small RNA was quantified by spectrophotometer (NanoDrop, Wilmington, USA). Twenty micrograms of small RNA was loaded per lane, and resolved on a denaturing 15% polyacrylamide gel containing 8 M urea at 20 mA; small RNA was transferred by electrophoresis from the gel to a positively charged nylon membrane (Millipore, Shanghai, China) by a semi-dry transfer apparatus (ATTA) at 200 mA for 2 h. After electroblotting, membranes were ultraviolet crosslinked (120 mJ, 30 s) and baked for 1 h at 80°C. DNA probes complementary to small RNA sequences were 5' end-labeled with γ-^32^P-ATP using a 5' DNA Labeling Kit (Shenergy Biocolor, Shanghai, China). The membrane was prehybridized in prehybridization solution (6 × SSC, 10 × Denhardt's solution, 0.2% SDS) at 64°C for 1 h. Membranes were hybridized in hybridization solution (6 × SSC, 5 × Denhardt's solution, 0.2% SDS) containing 1–5 × 10^6 ^cpm 5' end-labeled antisense probes for 18–24 h with gentle agitation at 28°C. Blots were washed three times for 5 min each at 28°C with 6 × SSC and 0.2% SDS, and once at 42°C for 10 min. After the final wash, blots were wrapped in plastic wrap and exposed to a phosphorimager screen (Amersham, Piscataway, USA) according to manufacturer instructions.

### Computational prediction of miRNA targets

Functions of the identified *B. mori *miRNAs were analyzed according to the functional conservation and the potential binding sites between miRNAs and the 3'UTR of responding mRNAs.

Based on the Tarbase database [59], we searched for the known targeted genes of *Drosophila *orthologs of identified miRNAs. Their *B. mori *homologs and their 3'UTR were subsequently obtained by the BLAST program. The *B. mori *miRNA targeted genes were predicted according to the binding between miRNAs and their complementary sites on the 3'UTR of the *B. mori *homologs by the RNAhybrid program [60].

In general, base pairing between miRNA and the complementary site on mRNA may provide a function prediction for miRNA. We calculated the potential binding sites between identified miRNAs and the 3'UTR of mRNAs of *B. mori *genes using the RNAhybrid program [60]; we used two settings to obtain specific binding sites. The first setting (P-value cutoff of 0.05, minimum free energy cutoff of -20.0 kcal/mol, maximum internal and bulge loop size of 1) was termed "hybrid11"; if maximum internal and bulge loop size was 2 but the seed region of miRNA could perfectly bind to 3'UTR of a gene (helix constraint of 2.7), we also considered this gene to be a potential miRNA-targeted gene (this setting was termed "hybridf22"). The functional annotation for predicted targeted genes were analyzed using Gene Ontology analysis [61]. *B. mori *genes were obtained from *B. mori *Genes Database constructed by us, and which includes 1909 genes. From these genes, 1671 3'UTRs were obtained and used for predicting potential targeted genes.

## Authors' contributions

PAH carried out the prediction and analysis of the miRNA and drafted the manuscript. ZMN performed the experiments, and carried out the analysis of miRNA and drafted the manuscript. JQC, JC, ZBL, QS and LYK performed the experiments. SPZ and XLG helped to analyze the data and draft the manuscript. XFW directed all the work of the manuscript. YFJ and YZZ conceived, and carried out the design and draft of the manuscript. All authors read and approved the final manuscript.

## Supplementary Material

Additional file 1 Northern blotting analysis of *B. mori *miRNAs. The data provided show the results of Northern blotting analysis for miRNAs. One table and three figures are in the file. The table shows the 21 plausible miRNAs identified by Northern blotting. Figure 1 displayed the result of Northern blotting analysis for *bmo-miR-277*. Figure 2 shows improvement of the detectable level of miRNA using methylated probes. Figure 3 shows the multi-alignment of the members of miRNA *let-7 *family. Click here for file

Additional file 2 Functional analysis of miRNAs in *B. mori *according to functional conservation between silkworm and fruit fly. The data provided show the detailed information regarding predicting targets of miRNAs in *B. mori*. Targets were predicted according to the binding between miRNAs and *B. mori *orthologs of known miRNA targets of fruit fly. Click here for file

Additional file 3 Functional analysis of miRNAs in *B. mori *according to binding sites between miRNAs and mRNAs. The data provided show the detailed information for miRNA-targeted genes predicted according to binding sites on 3'UTR of mRNAs. There are six sheets in the EXCEL file, including hybrid11, hybridf22, combination with no redundancy, statistics of binding sites, Go analysis, and binding sites of *profilin*. Sheet hybrid11 shows detailed information of predicted target genes using setting hybrid11; sheet hybridf22 shows detailed information of predicted target genes using setting hybridf22; sheet 3, named "combination with no redundancy", shows the results for the sheet hybrid11 and hybridf22 after removing redundancy; sheet 4, named "statistics of binding sites", shows the results of statistic analysis for all genes that were found to have more than one binding site; sheet 5, named "Go analysis", shows the results of Go analysis of predicted targeted genes; and sheet 6, named "binding sites of *profilin*", shows information of the potential binding sites between the *B. mori profilin *gene and miRNAs. Click here for file

Additional file 4 Phylogeny trees for each of miRNA families. The data provided show the phylogeny trees for each of miRNA families in *B. mori*. Click here for file

Additional file 5 Detailed information of all the 46 identified miRNAs in *B. mori*. The data provided show the detailed information of all the 46 identified miRNAs in *B. mori*, including sequences of the pre-miRNA and mature miRNA, the complementary region of miRNAs, the secondary structure of pre-miRNA, and the minimum energy. Click here for file

## References

[B1] Ambros V (2003). MicroRNA pathways in flies and worms: growth, death, fat, stress, and timing. Cell.

[B2] Ambros V (2004). The functions of animal microRNAs. Nature.

[B3] Lim LP, Lau NC, Weinstein EG, Abdelhakim A, Yekta S, Rhoades MW, Burge CB, Bartel DP (2003). The microRNAs of Caenorhabditis elegans. Genes Dev.

[B4] Carrington JC, Ambros V (2003). Role of microRNAs in plant and animal development. Science.

[B5] Lee RC, Feinbaum RL, Ambros V (1993). The C. elegans heterochronic gene lin-4 encodes small RNAs with antisense complementarity to lin-14. Cell.

[B6] Reinhart BJ, Slack FJ, Basson M, Pasquinelli AE, Bettinger JC, Rougvie AE, Horvitz HR, Ruvkun G (2000). The 21-nucleotide let-7 RNA regulates developmental timing in Caenorhabditis elegans. Nature.

[B7] Lagos-Quintana M, Rauhut R, Lendeckel W, Tuschl T (2001). Identification of novel genes coding for small expressed RNAs. Science.

[B8] Lau NC, Lim LP, Weinstein EG, Bartel DP (2001). An abundant class of tiny RNAs with probable regulatory roles in Caenorhabditis elegans. Science.

[B9] Lee RC, Ambros V (2001). An extensive class of small RNAs in Caenorhabditis elegans. Science.

[B10] Grad Y, Aach J, Hayes GD, Reinhart BJ, Church GM, Ruvkun G, Kim J (2003). Computational and experimental identification of C. elegans microRNAs. Mol Cell.

[B11] Lai EC, Tomancak P, Williams RW, Rubin GM (2003). Computational identification of Drosophila microRNA genes. Genome Biol.

[B12] Wang XJ, Reyes JL, Chua NH, Gaasterland T (2004). Prediction and identification of Arabidopsis thaliana microRNAs and their mRNA targets. Genome Biol.

[B13] Jones-Rhoades MW, Bartel DP (2004). Computational identification of plant microRNAs and their targets, including a stress-induced miRNA. Mol Cell.

[B14] Bonnet E, Wuyts J, Rouze P, Peer Y Van de (2004). Evidence that microRNA precursors, unlike other non-coding RNAs, have lower folding free energies than random sequences. Bioinformatics.

[B15] Yoon S, De Micheli G (2005). Prediction of regulatory modules comprising microRNAs and target genes. Bioinformatics.

[B16] Wang X, Zhang J, Li F, Gu J, He T, Zhang X, Li Y (2005). MicroRNA identification based on sequence and structure alignment. Bioinformatics.

[B17] Nam JW, Shin KR, Han J, Lee Y, Kim VN, Zhang BT (2005). Human microRNA prediction through a probabilistic co-learning model of sequence and structure. Nucleic Acids Res.

[B18] Dezulian T, Remmert M, Palatnik JF, Weigel D, Huson DH (2006). Identification of plant microRNA homologs. Bioinformatics.

[B19] Tong CZ, Jin YF, Zhang YZ (2006). Computational prediction of microRNA genes in silkworm genome. J Zhejiang Univ Sci B.

[B20] Helvik SA, Snove O, Saetrom P (2007). Reliable prediction of Drosha processing sites improves microRNA gene prediction. Bioinformatics.

[B21] Griffiths-Jones S, Grocock RJ, van Dongen S, Bateman A, Enright AJ (2006). miRBase: microRNA sequences, targets and gene nomenclature. Nucleic Acids Res.

[B22] Griffiths-Jones S (2004). The microRNA Registry. Nucleic Acids Res.

[B23] Ambros V, Bartel B, Bartel DP, Burge CB, Carrington JC, Chen X, Dreyfuss G, Eddy SR, Griffiths-Jones S, Marshall M (2003). A uniform system for microRNA annotation. RNA.

[B24] Arteaga-Vazquez M, Caballero-Perez J, Vielle-Calzada JP (2006). A family of microRNAs present in plants and animals. Plant Cell.

[B25] Xia Q, Zhou Z, Lu C, Cheng D, Dai F, Li B, Zhao P, Zha X, Cheng T, Chai C (2004). A draft sequence for the genome of the domesticated silkworm (Bombyx mori). Science.

[B26] Wang J, Xia Q, He X, Dai M, Ruan J, Chen J, Yu G, Yuan H, Hu Y, Li R (2005). SilkDB: a knowledgebase for silkworm biology and genomics. Nucleic Acids Res.

[B27] Schwarz DS, Hutvagner G, Du T, Xu Z, Aronin N, Zamore PD (2003). Asymmetry in the assembly of the RNAi enzyme complex. Cell.

[B28] Gregory RI, Chendrimada TP, Cooch N, Shiekhattar R (2005). Human RISC couples microRNA biogenesis and posttranscriptional gene silencing. Cell.

[B29] Velkey JM, Slawny NA, Gratsch TE, O'Shea KS (2006). Gene silencing using RNA interference in embryonic stem cells. Methods Mol Biol.

[B30] Bartel DP (2004). MicroRNAs: genomics, biogenesis, mechanism, and function. Cell.

[B31] Rana TM (2007). Illuminating the silence: understanding the structure and function of small RNAs. Nat Rev Mol Cell Biol.

[B32] Zhang B, Wang Q, Pan X (2007). MicroRNAs and their regulatory roles in animals and plants. J Cell Physiol.

[B33] Behura SK (2007). Insect microRNAs: Structure, function and evolution. Insect Biochem Mol Biol.

[B34] Denli AM, Tops BB, Plasterk RH, Ketting RF, Hannon GJ (2004). Processing of primary microRNAs by the Microprocessor complex. Nature.

[B35] Kloosterman WP, Wienholds E, Ketting RF, Plasterk RH (2004). Substrate requirements for let-7 function in the developing zebrafish embryo. Nucleic Acids Res.

[B36] Liu S, Xia Q, Zhao P, Cheng T, Hong K, Xiang Z (2007). Characterization and expression patterns of let-7 microRNA in the silkworm (Bombyx mori). BMC Dev Biol.

[B37] Zuker M (2003). Mfold web server for nucleic acid folding and hybridization prediction. Nucleic Acids Res.

[B38] Grun D, Wang YL, Langenberger D, Gunsalus KC, Rajewsky N (2005). microRNA target predictions across seven Drosophila species and comparison to mammalian targets. PLoS Comput Biol.

[B39] Sethupathy P, Corda B, Hatzigeorgiou AG (2006). TarBase: A comprehensive database of experimentally supported animal microRNA targets. RNA.

[B40] Roder K, Hung MS, Lee TL, Lin TY, Xiao H, Isobe KI, Juang JL, Shen CJ (2000). Transcriptional repression by Drosophila methyl-CpG-binding proteins. Mol Cell Biol.

[B41] Guimaraes-Sternberg C, Meerson A, Shaked I, Soreq H (2006). MicroRNA modulation of megakaryoblast fate involves cholinergic signaling. Leuk Res.

[B42] Majlessi M, Nelson NC, Becker MM (1998). Advantages of 2'-O-methyl oligoribonucleotide probes for detecting RNA targets. Nucleic Acids Res.

[B43] Tanzer A, Stadler PF (2004). Molecular evolution of a microRNA cluster. J Mol Biol.

[B44] Aravin AA, Lagos-Quintana M, Yalcin A, Zavolan M, Marks D, Snyder B, Gaasterland T, Meyer J, Tuschl T (2003). The small RNA profile during Drosophila melanogaster development. Dev Cell.

[B45] Seitz H, Royo H, Bortolin ML, Lin SP, Ferguson-Smith AC, Cavaille J (2004). A large imprinted microRNA gene cluster at the mouse Dlk1-Gtl2 domain. Genome Res.

[B46] Valencia-Sanchez MA, Liu J, Hannon GJ, Parker R (2006). Control of translation and mRNA degradation by miRNAs and siRNAs. Genes Dev.

[B47] Lee Y, Jeon K, Lee JT, Kim S, Kim VN (2002). MicroRNA maturation: stepwise processing and subcellular localization. EMBO J.

[B48] Stark A, Brennecke J, Russell RB, Cohen SM (2003). Identification of Drosophila MicroRNA targets. PLoS Biol.

[B49] Sethupathy P, Megraw M, Hatzigeorgiou AG (2006). A guide through present computational approaches for the identification of mammalian microRNA targets. Nat Methods.

[B50] Joglekar MV, Parekh VS, Mehta S, Bhonde RR, Hardikar AA (2007). MicroRNA profiling of developing and regenerating pancreas reveal post-transcriptional regulation of neurogenin3. Dev Biol.

[B51] Asangani IA, Rasheed SA, Nikolova DA, Leupold JH, Colburn NH, Post S, Allgayer H (2007). MicroRNA-21 (miR-21) post-transcriptionally downregulates tumor suppressor Pdcd4 and stimulates invasion, intravasation and metastasis in colorectal cancer. Oncogene.

[B52] Weaver DB, Anzola JM, Evans JD, Reid JG, Reese JT, Childs KL, Zdobnov EM, Samanta MP, Miller J, Elsik CG (2007). Computational and transcriptional evidence for microRNAs in the honey bee genome. Genome Biol.

[B53] Lai EC (2002). Micro RNAs are complementary to 3' UTR sequence motifs that mediate negative post-transcriptional regulation. Nat Genet.

[B54] Clarke SR, Staiger CJ, Gibbon BC, Franklin-Tong VE (1998). A potential signaling role for profilin in pollen of Papaver rhoeas. Plant Cell.

[B55] miRBase website. http://microrna.sanger.ac.uk/sequences/index.shtml.

[B56] Beijing Genomics Institute website. http://silkworm.genomics.org.cn/.

[B57] Gao X, Gulari E, Zhou X (2004). In situ synthesis of oligonucleotide microarrays. Biopolymers.

[B58] Bolstad BM, Irizarry RA, Astrand M, Speed TP (2003). A comparison of normalization methods for high density oligonucleotide array data based on variance and bias. Bioinformatics.

[B59] Tarbase website. http://www.diana.pcbi.upenn.edu/tarbase.html.

[B60] Rehmsmeier M, Steffen P, Hochsmann M, Giegerich R (2004). Fast and effective prediction of microRNA/target duplexes. RNA.

[B61] Gene Ontology analysis website. http://goblet.molgen.mpg.de/cgi-bin/goblet/webapp-goblet.cgi?menu=Form.

