# Supplementary material for: Identification and characteristics of microRNAs from Bombyx mori
Source: BMC Genomics. 2008 May 28;9:248. doi: 10.1186/1471-2164-9-248 (PMC2435238; doi:10.1186/1471-2164-9-248)
Supplement: Additional file 1 — Northern blotting analysis of B. mori miRNAs. The data provided show the results of Northern blotting analysis for miRNAs. One table and three figures are in the file. The table shows the 21 plausible miRNAs identified by Northern blotting. Figure 1 displayed the result of Northern blotting analysis for bmo-miR-277. Figure 2 shows improvement of the detectable level of miRNA using methylated probes. Figure 3 shows the multi-alignment of the members of miRNA let-7 family. [file 1471-2164-9-248-S1.doc]

**Additional file 1**

**Table 1**. The 21 plausible miRNAs identified by northern blot.

| miRNAs | sequences | Northern blot identification |
| --- | --- | --- |
| *bmo-mir-228* | AAUGGCACUGCAUGAAUUCACGG | no |
| *bmo-mir-243* | CGGUACGAUCGCGGCGGGAUAUC | no |
| *bmo-mir-252* | UAAGUAGUAGUGCCGCAGGUAAC | no |
| *bmo-mir-58* | UGAGAUCGUUCAGUACGGCAAU | no |
| *bmo-mir-81* | UGAGAUCAUCGUGAAAGCUAGU | no |
| *bmo-mir-100* | AACCCGUAAAUCCGAACUUGUG | yes |
| *bmo-mir-276b* | UAGGAACUUAAUACCGUGCUCU | yes |
| *bmo-mir-281b** | AAGAGAGCUGUCCGUCGACAGU | yes |
| *bmo-let-7b* | AGAGGUAGUAGGUUGCAUAGU | yes |
| *bmo-let-7c* | UGAGGUAGGAGGUUGUAUAGU | yes |
| *bmo-let-7d* | UGAGGUAGUAGAUUGUAUAGUU | yes |
| *bmo-mir-289* | UAAAUAUUUAAGUGGAGCCUGCGACU | yes |
| *bmo-mir-183* | UAUGGCACUGGUAGAAUUCACUG | no |
| *bmo-mir-187* | UCGUGUCUUGUGUUGCAGCCG | no |
| *bmo-mir-206* | UGGAAUGUAAGGAAGUGUGUGG | no |
| *bmo-mir-214* | ACAGCAGGCACAGACAGGCAG | no |
| *bmo-mir-23* | AUCACAUUGCCAGGGAUUACC | no |
| *bmo-mir-26* | UUCAAGUAAUUCAGGAUAGGUU | no |
| *bmo-mir-489* | AGUGACAUCACAUAUACGGCAGC | no |
| *bmo-mir-608* | AGGGGUGGUGUUGGGACAGCUCCGU | no |
| *bmo-mir-638* | AGGGAUCGCGGGCGGGUGGCGGCCU | no |

**Larva Pupa Moth**

**101nt**

**23nt**

**Precursor**

***bmo-mir-277***

**5S rRNA**

Figure 1. Northern blot analysis of *bmo-mir-277*. *bmo-mir-277* was only expressed in moth and could not be detected in larva and pupa. The 101nt precursor of the miRNA was also detected.


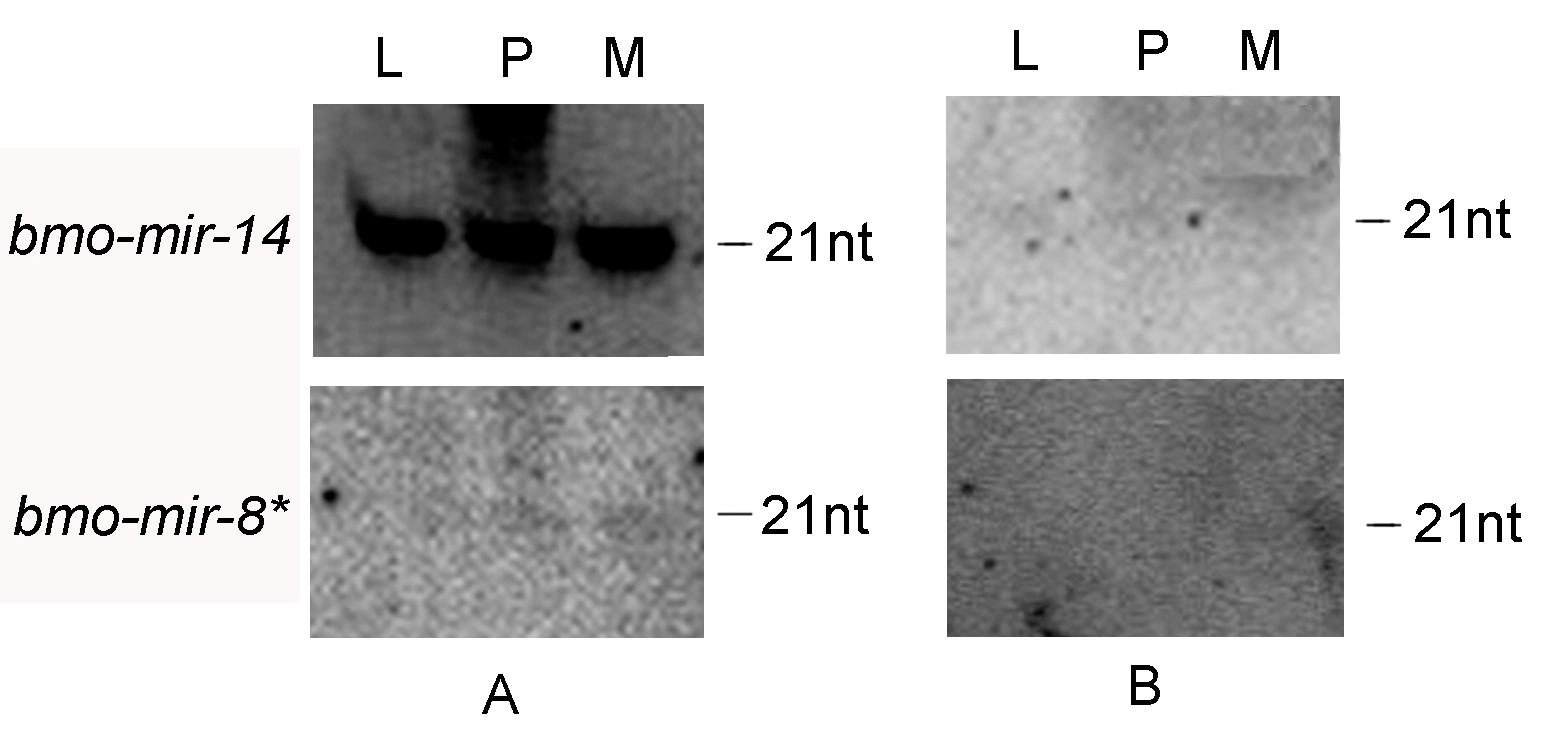


Figure 2. Improvement of detectable level of miRNA using methylation of probe. A, northern blot analysis using methylated probe: *bmo-mir-14*: TAGGAGAGAGAAAAAGACTGA ; *bmo-mir-8**: TAATGCTGCCCGGTAAGATGC; the methylated base was boxed. B, northern blot analysis using normal probe. L: larva; P: pupa; M: moth.


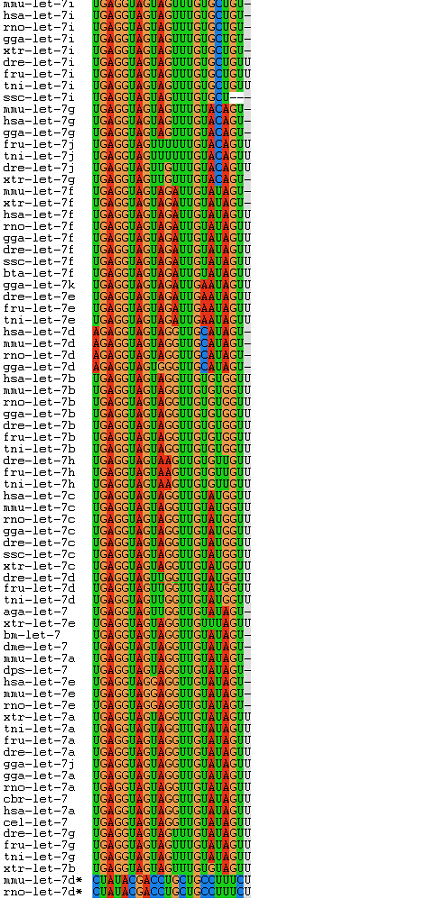


Figure 3. Multialignment of the members of miRNA *let-7* family
