# Supplementary material for: Identification and characteristics of microRNAs from Bombyx mori
Source: BMC Genomics. 2008 May 28;9:248. doi: 10.1186/1471-2164-9-248 (PMC2435238; doi:10.1186/1471-2164-9-248)
Supplement: Additional file 2 — Functional analysis of miRNAs in B. mori according to functional conservation between silkworm and fruit fly. The data provided show the detailed information regarding predicting targets of miRNAs in B. mori. Targets were predicted according to the binding between miRNAs and B. mori orthologs of known miRNA targets of fruit fly. [file 1471-2164-9-248-S2.doc]

Table 1 Prediction for the functions of miRNAs in *Bombyx mori* *

| *Bombyx mori* miRNA | *Drosophila* ortholgs  (DO) | Known targeted genes of DO  (KTGDO) | Functional Annotation for KTGDO | *Bombyx mori* Homologs of KTGDO as potential miRNA targeted genes  (BmHKTGDO) | minimum free energy (mfe) of the binding between miRNAs and its complementary sites in 3’UTR of BmHKTGDO |
| --- | --- | --- | --- | --- | --- |
| bmo-mir-2a  bmo-mir-2b  bmo-mir-13a*  bmo-mir-13b | dme-mir-2 | Grim  (Uniprot:Q24570) | Activator of apoptosis, independent of rpr and W, that acts on the effector, Dredd. Expression coincides with the onset of programmed cell death (PCD) at all stages of embryonic development. | *Bombyx* homolog of P-element somatic inhibitor  NM_001110343 | mfe: -23.5 kcal/mol  p-value: 0.220850  mfe: -21.4 kcal/mol  p-value: 0.404324  mfe: -20.5 kcal/mol  p-value: 0.485591  mfe: -24.7 kcal/mol  p-value: 0.146465 |
|  |  | HLHmdelta  (Uniprot:Q01071) | Transcriptional repressor of genes that require a bHLH protein for their transcription. May serve as a transcriptional regulator of the Achaete-scute complex (AS-C) genes. Contributes to the neural-epidermal lineage decision during early neurogenesis. Part of the Notch signaling pathway. | UniGene:Bmo.1984 | mfe: -19.4 kcal/mol  p-value: 0.351294  mfe: -20.3 kcal/mol  p-value: 0.262035  mfe: -20.4 kcal/mol  p-value: 0.236167  mfe: -17.9 kcal/mol  p-value: 0.530808 |
| bmo-mir-7 | dmo-mir-7 | HLHm3  (Uniprot:Q01068)  HLHmgamma  (Uniprot:Q01070)  HLHm5  (Uniprot:P13096) | These targeted genes are homologs of HLHmdelta and have the same or similar functions as HLHmdelta. | UniGene:Bmo.1984 | mfe: -18.5 kcal/mol  p-value: 0.482372 |
|  |  | m4  (Uniprot:P13095)  TOM  (Uniprot:Q9U4W9) | Part of the Notch signaling pathway. | UniGene:Bmo.4220 | mfe: -28.2 kcal/mol  p-value: 0.016022 |
|  |  | YAN (Uniprot:Q01842) | Negative regulator of photoreceptor development that acts antagonistically to the proneural signal mediated by RAS. It acts upstream of SINA to inhibit R7 development. | UniGene:Bmo.4224 | mfe: -25.0 kcal/mol  p-value: 0.080414 |
| bmo-mir-79 | dmo-mir-79 | m4  (Uniprot:P13095)  TOM  (Uniprot:Q9U4W9) | Part of the Notch signaling pathway. | UniGene:Bmo.4220 | mfe: -17.6 kcal/mol  p-value: 0.623192 |
| bmo-mir-14 | dmo-mir-14 | Drice (Uniprot:O01382) | Involved in the activation cascade of caspases responsible for apoptosis execution. Acts downstream of rpr. Cleaves baculovirus p35 and lamin DmO in vitro. | caspase-1  NM_001043585 | mfe: -13.8 kcal/mol  p-value: 0.953081 |
| bmo-mir-34 | dmo-mir-34 | Eip74EF (Uniprot:P11536) | The expression of this protein is developmentally regulated and is correlated with the 20-OH-ecdysone induced activity of puff 74EF. | Transcription factor E74 isoform A  NM_001043979 | mfe: -27.6 kcal/mol  p-value: 0.044975 |
| bmo-mir-279 | dmo-mir-279 | SP555  (Uniprot:Q9V3U3) | Intracellular signaling cascade | UniGene:Bmo2301 | mfe: -14.2 kcal/mol  p-value: 0.945715 |
| bmo-bantam | dmo-bantam | W  (Uniprot:Q24106) | Activator of apoptosis, with grim and rpr, that acts on the effector, Dredd. Sems to act genetically upstream of baculoviral anti-apoptotic p35. | ribonucleic acid binding protein S1  NP_001040150 | mfe: -20.1 kcal/mol  p-value: 0.344833 |
| bmo-let-7 | dme-let-7 | Ab  (Uniprot:Q24174) | Expression is vital for development. In embryos, muscle specific expression required for segmental nerve b (SNb) motoneuron target recognition with ventral longitudinal muscles. Has a role in establishing and maintaining embryonic muscle attachments, adult sensory cell formation (macrochaetae) and morphogenesis of adult appendages (legs, antenna aristae, male external genitalia). May be involved in transcriptional regulation. | CK535854 | mfe: -20.7 kcal/mol  p-value: 0.057985 |
| bmo-mir-133 | mir-133(Mus musculus ) | SRF  (UniProt:Q9JM73) | SRF is a transcription factor that binds to the serum response element (SRE), a short sequence of dyad symmetry located 300 bp to the 5' of the site of transcription initiation of some genes (such as FOS) (By similarity). Required for cardiac differentation and maturation. | myocyte enhancer factor-2  NP_001036905 | mfe: -19.2 kcal/mol  p-value: 0.521678 |
|  | mir-133(Mus musculus ) | Ptbp2  (Uniprot:Q91Z31) | RNA-binding protein which binds to intronic polypyrimidine tracts and mediates negative regulation of exons splicing. May antagonize in a tissue-specific manner the ability of NOVA1 to activate exon selection. Beside its function in pre-mRNA splicing, plays also a role in the regulation of translation. | TIA-1 like protein  BAB16700 | mfe: -25.5 kcal/mol  p-value: 0.063250 |

*The detailed information for the binding between miRNAs and their potential targeted genes is listed as follows:

**miRNA : bmo-let-7**

length: 19

**target: CK535854**

length: 116

mfe: -20.7 kcal/mol

p-value: 0.057985

position 54

target 5' A AU UG G 3'

UGUAUA GCCU U CGUC

AUAUGU UGGA A GUAG

miRNA 3' G UG U 5'

**miRNA : bmo-mir-2a**

length: 23

**target: NM_00111034**3

length: 1252

mfe: -23.5 kcal/mol

p-value: 0.220850

position 15

target 5' G C G C 3'

GUCGAG GC GGCUGU

UAGUUU CG CCGACA

miRNA 3' CGAG A CUAU 5'

**miRNA : bmo-mir-2b**

length: 23

**target: NM_001110343**

length: 1252

mfe: -21.4 kcal/mol

p-value: 0.404324

position 444

target 5' U A U C 3'

UUC AACG GGCUG UGUGGU

GAG UUGU UCGAC ACACUA

miRNA 3' U U CG U 5'

**miRNA : bmo-mir-13a***

length: 21

**target: NM_001110343**

length: 1252

mfe: -20.5 kcal/mol

p-value: 0.485591

position 333

target 5' A GA C 3'

UUCACU UGUUU GACAG

AAGUGG GCGAA CUGUC

miRNA 3' A CG A C 5'

**miRNA : bmo-mir-13b**

length: 22

**target: NM_001110343**

length: 1252

mfe: -24.7 kcal/mol

p-value: 0.146465

position 445

target 5' U C U 3'

UCAAA GUGGCUGUG

AGUUU UACCGACAC

miRNA 3' GAGC U UAU 5'

**miRNA : bmo-mir-2a**

length: 23

**target: UniGene:Bmo.1984**

length: 385

mfe: -19.4 kcal/mol

p-value: 0.351294

position 202

target 5' G U AAU U 3'

UAUC AAGUU UUGUGAUA

GUAG UUCGA GACACUAU

miRNA 3' CGA U CC 5'

**miRNA : bmo-mir-2b**

length: 23

**target: UniGene:Bmo.1984**

length: 385

mfe: -20.3 kcal/mol

p-value: 0.262035

position 12

target 5' G C G C 3'

GAUGGAGC GGC GA

UUGUUUCG CCG CU

miRNA 3' UGAG A ACA AU 5'

**miRNA : bmo-mir-13a***

length: 21

**target: UniGene:Bmo.1984**

length: 385

mfe: -20.4 kcal/mol

p-value: 0.236167

position 4

target 5' A G GA GGAGCC A 3'

CG UGCCG U GGCGG

GU GCGGC A CUGUC

miRNA 3' AAA G GA A C 5'

**miRNA : bmo-mir-13b**

length: 22

**target: UniGene:Bmo.1984**

length: 385

mfe: -17.9 kcal/mol

p-value: 0.530808

position 305

target 5' A AUUUCACAUUUA A 3'

UCGUUAAAAG UGUGA

AGCAGUUUUU ACACU

miRNA 3' G ACCG AU 5'

**miRNA : bmo-mir-7**

length: 25

**target: UniGene:Bmo.1984**

length: 385

mfe: -18.5 kcal/mol

p-value: 0.482372

position 20

target 5' C CCCGGCUCCGCG A GU G 3'

CGGCGGA GUCAC UCU UCCA

GUUGUUU UAGUG AGA AGGU

miRNA 3' AUC AUG 5'

**miRNA : bmo-mir-7**

length: 25

**target: UniGene:Bmo.4220**

length: 408

mfe: -28.2 kcal/mol

p-value: 0.016022

position 10

target 5' G C G A 3'

CGA CAGGAUCA U GUCUUCCGUG

GUU GUUUUAGU A CAGAAGGUAU

miRNA 3' G U G 5'

**miRNA : bmo-mir-7**

length: 25

**target: UniGene:Bmo4224**

length: 613

mfe: -25.0 kcal/mol

p-value: 0.080414

position 95

target 5' A GN G 3'

UA CAAGAUU UAGUCUUCCG

GU GUUUUAG AUCAGAAGGU

miRNA 3' U UG AUG 5'

**miRNA : bmo-mir-79**

length: 25

**target: UniGene:Bmo.4220**

length: 408

mfe: -17.6 kcal/mol

p-value: 0.623192

position 70

target 5' U G A 3'

GUGAU UUAGCUUUA

CAUUA GAUCGAAAU

miRNA 3' ACGAAAC ACUU 5'

**miRNA : bmo-mir-14**

length: 20

**target: NM_001043585**

length: 299

mfe: -13.8 kcal/mol

p-value: 0.953081

position 183

target 5' U UGU C 3'

GAGA AAGAUU

CUCU UUCUGA

miRNA 3' UCCUCU UU CU 5'

**miRNA : bmo-mir-34**

length: 24

**target: NM_001043979**

length: 911

mfe: -27.6 kcal/mol

p-value: 0.044975

position 313

target 5' G G GC G GA UG G 3'

ACGCGGCC AGUU GA U GC UGUC

UGUGUUGG UCGA UU G UG ACGG

miRNA 3' G UG 5'

**miRNA : bmo-mir-279**

length: 18

**target: UniGene:Bmo.2301**

length: 396

mfe: -14.2 kcal/mol

p-value: 0.945715

position 78

target 5' C CA AAGCU CGCAGC U 3'

GAG UG GAUCUA GUCG

CUC AC CUAGAU CAGU

miRNA 3' A AC 5'

**miRNA : bmo-bantam**

length: 22

**target: NP_001040150**

length: 543

mfe: -20.1 kcal/mol

p-value: 0.344833

position 153

target 5' A C A 3'

AGUUUUCGUAAUGA CU

UCGAAAGUGUUACU GA

miRNA 3' UAA A GU 5'

**miRNA : bmo-mir-133**

length: 21

**target: NP_001036905**

length: 755

mfe: -19.2 kcal/mol

p-value: 0.521678

position 2

target 5' A A CUA CCUUC U C 3'

UAGCU GG UGAGG GG CC

GUCGA CC ACUUC CC GG

miRNA 3' A C U UU 5'

**miRNA : bmo-mir-133**

length: 21

**target: BAB16700**

length: 684

mfe: -25.5 kcal/mol

p-value: 0.063250

position 279

target 5' C U U C C G 3'

CAG CUG UU G AAGGGGACU

GUC GAC AA C UUCCCCUGG

miRNA 3' C UU 5'
