# Supplementary material for: Identification and characteristics of microRNAs from Bombyx mori
Source: BMC Genomics. 2008 May 28;9:248. doi: 10.1186/1471-2164-9-248 (PMC2435238; doi:10.1186/1471-2164-9-248)
Supplement: Additional file 4 — Phylogeny trees for each of miRNA families. The data provided show the phylogeny trees for each of miRNA families in B. mori. [file 1471-2164-9-248-S4.doc]

**Additional file 4**

The phylogeny tree for each of miRNA family.

bantam

let-7

mir-1

mir-2

mir-7

mir-8

mir-9

mir-10

mir-13

mir-14

mir-31

mir-34

mir-46

mir-71

mir-79

mir-87

mir-133

mir-184

mir-210

mir-263

mir-275

mir-276

mir-277

mir-279

mir-281

mir-282

mir-283

mir-305

mir-307

mir-317
