# Supplementary material for: Identification and characteristics of microRNAs from Bombyx mori
Source: BMC Genomics. 2008 May 28;9:248. doi: 10.1186/1471-2164-9-248 (PMC2435238; doi:10.1186/1471-2164-9-248)

**Additional file 5**

**detailed information of all the 46 identified miRNAs in *Bombyx mori .***

The mature miRNA sequences were written in lowercase letters. The 13 counter-strand miRNA*s were indicated as asterisk.


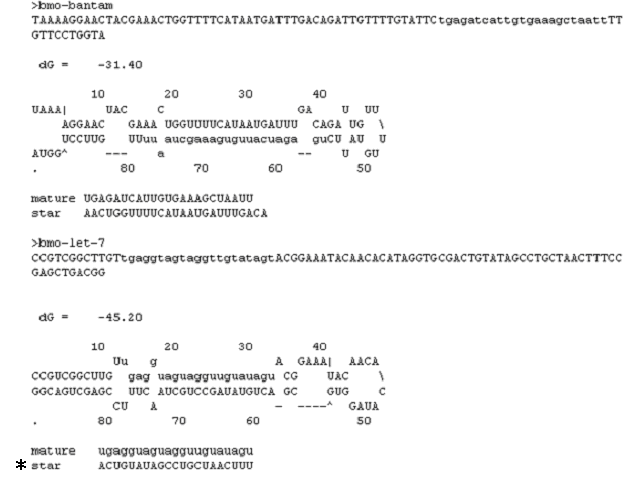


*


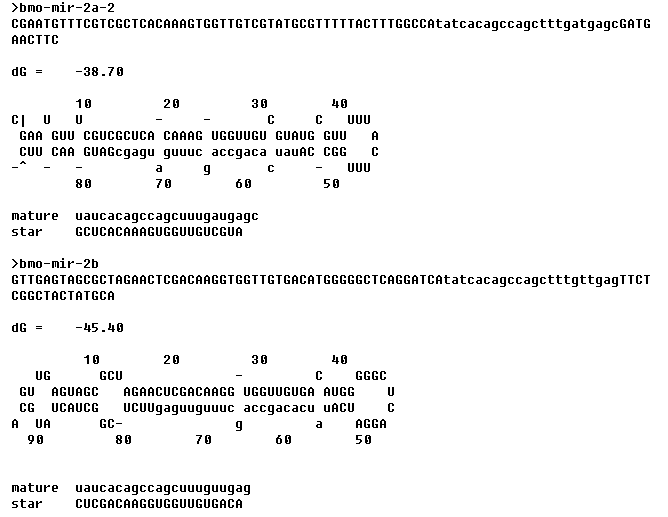


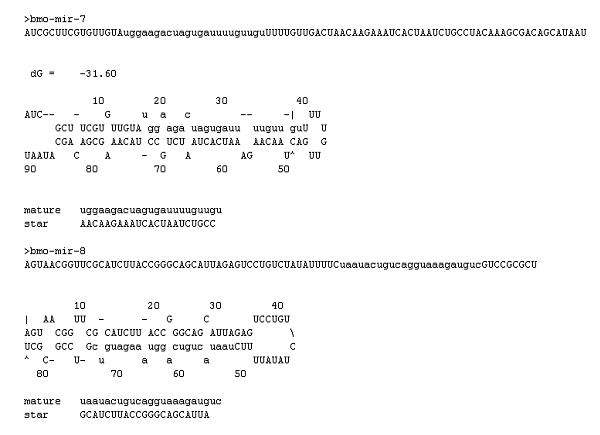


*

*

*


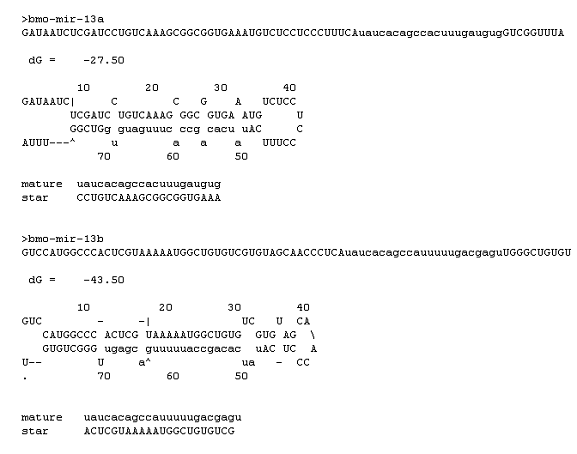


*

bmo-mir-13a can’t be detected by experimental methods, but bmo-mir-13a* is detected by microarray.


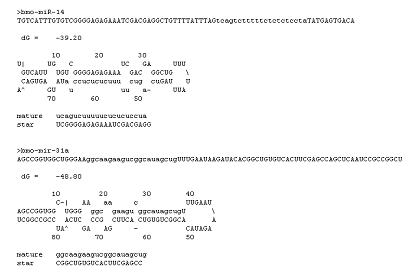


**>bmo-mir-14**


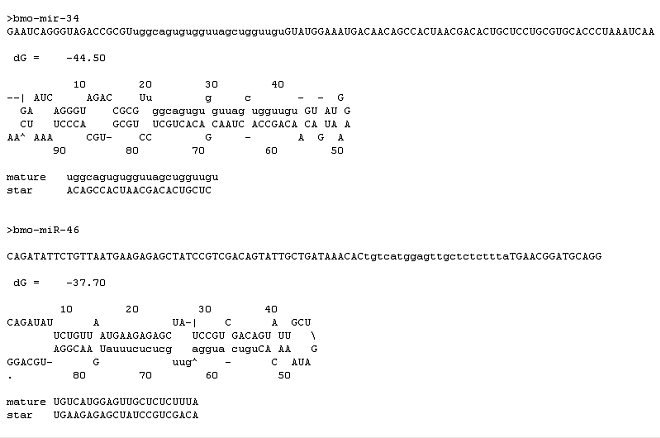


*


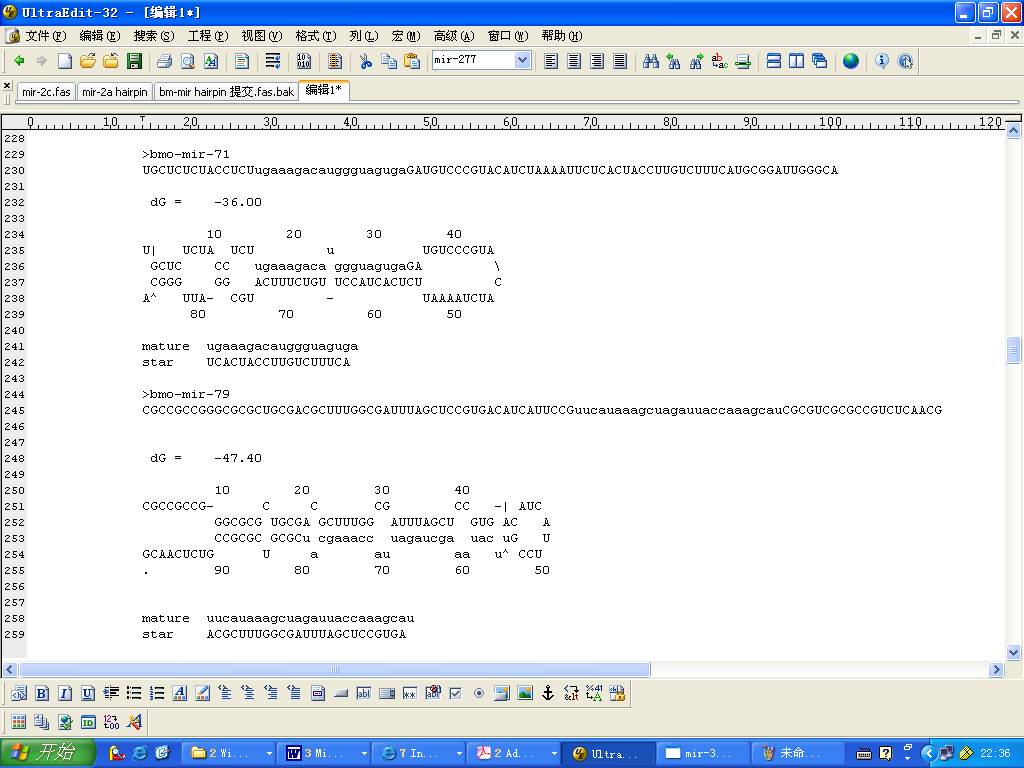


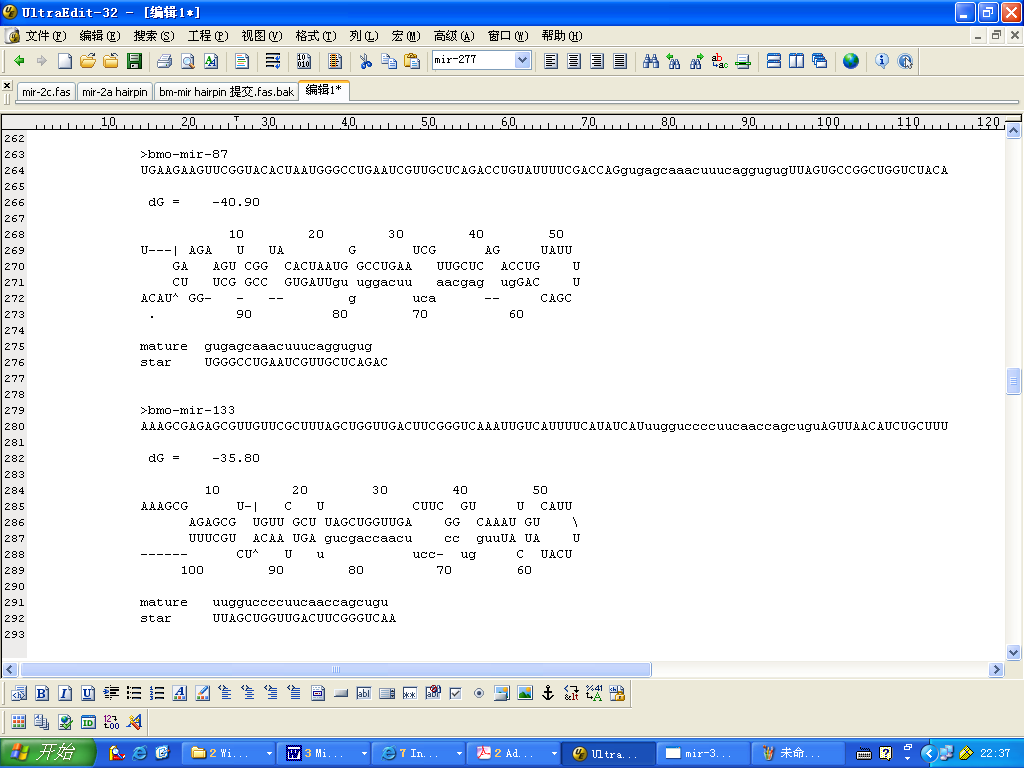


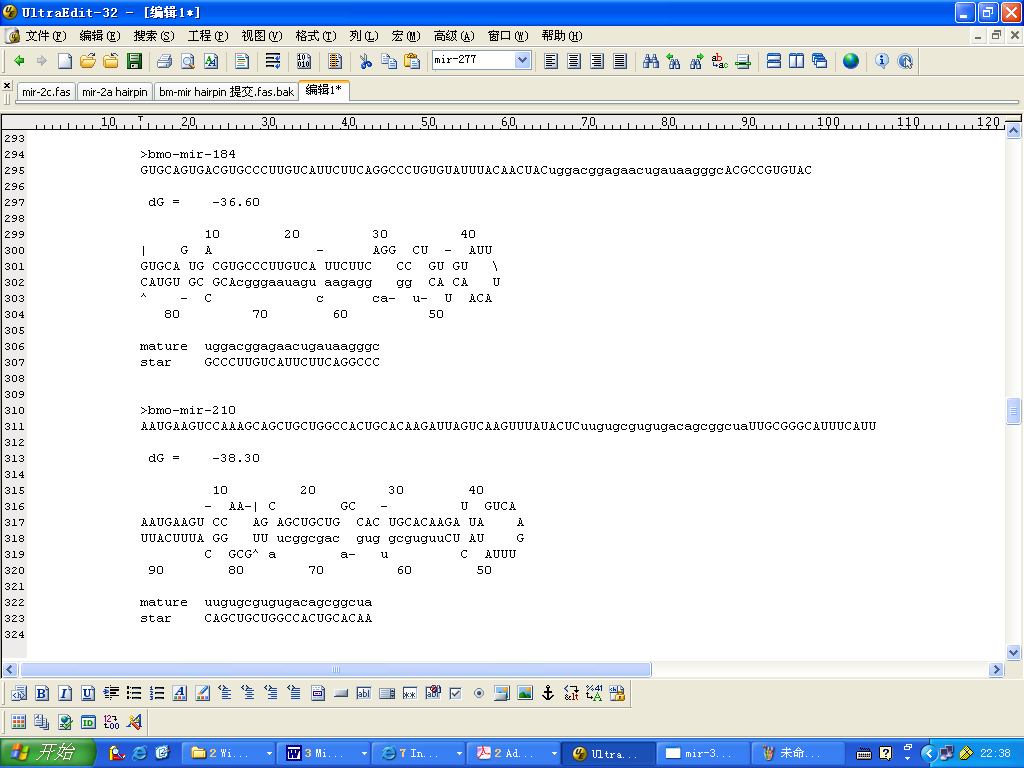


*


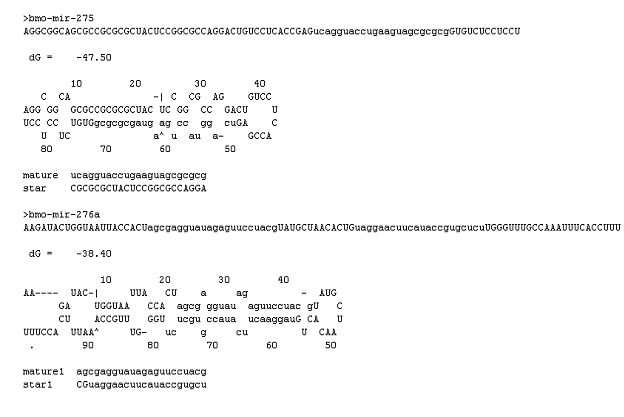


*

*

*

*


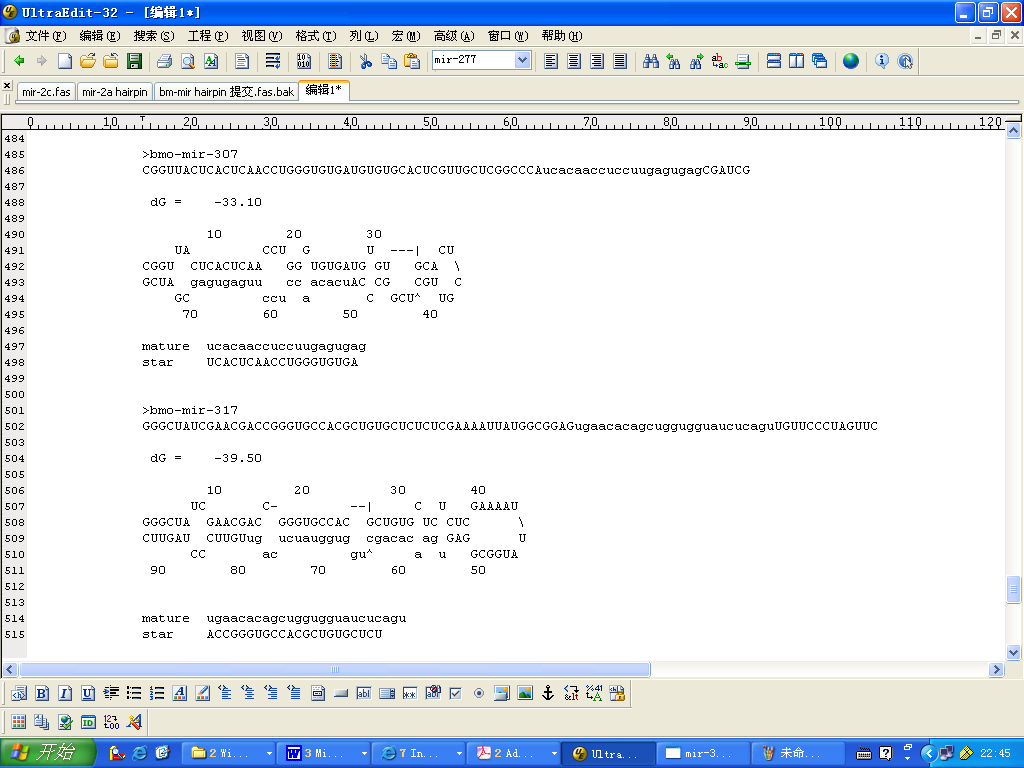

Supplement: Additional file 5 — Detailed information of all the 46 identified miRNAs in B. mori. The data provided show the detailed information of all the 46 identified miRNAs in B. mori, including sequences of the pre-miRNA and mature miRNA, the complementary region of miRNAs, the secondary structure of pre-miRNA, and the minimum energy. [file 1471-2164-9-248-S5.doc]
